# Integrated Network Pharmacology, Molecular Docking, and Experimental Validation Elucidating the Therapeutic Mechanism of *Idesia polycarpa* Crude Oil in Aluminum Chloride‐Induced Alzheimer's Rat Models

**DOI:** 10.1002/fsn3.71702

**Published:** 2026-04-20

**Authors:** Weijie Chang, Yaobing Chen, Jianquan Kan, Xiufang Huang, Kai Luo

**Affiliations:** ^1^ College of Biological and Food Engineering Hubei Minzu University Enshi Hubei China; ^2^ Hubei Key Laboratory of Biologic Resources Protection and Utilization Hubei Minzu University Enshi Hubei China; ^3^ College of Food Science Southwest University Chongqing China

**Keywords:** Alzheimers disease, gut microbiota, *Idesia polycarpa* crude oil, molecular docking, network pharmacology

## Abstract

While mounting evidence points to a potential link between industrial aluminum exposure and neurodegenerative diseases like Alzheimer's disease (AD), the precise intervention strategies remain an area of active research. This study proposes a “multi‐target synergy and dose threshold control” exploratory framework for evaluating *Idesia polycarpa* crude oil (IPCO) in an aluminum‐induced AD model. An integrated analytical approach employing GC–MS and network pharmacology was used to identify three candidate core components—(Z,Z)‐9,12‐octadecadienoic acid, Beta‐amyrin, and 2,4‐di‐tert‐butylphenol—that were computationally predicted to influence a network of 35 ad‐related pathways (e.g., Calcium and PPAR signaling) via eight potential key targets (including *PTGS2*, *PPARG*, and *AKT1*). In vivo experiments revealed a dose‐dependent modulation of AD‐related pathology following IPCO intervention. The high‐dose group showed the most marked improvements in several therapeutic markers, including reduced aluminum load, an anti‐inflammatory shift in cytokine levels (elevated IL‐10, decreased IL‐4, IL‐6, IL‐1β, and TNF‐α), and remodeling of the gut microbiota characterized by an increase in putative short‐chain fatty acid (SCFA)‐producing genera such as the *[Eubacterium]‐xylanophilum‐group* and *NK4A214‐group* (*Firmicutes*). Paradoxically, this same high dose was associated with a decline in spatial cognitive performance. This biphasic effect may be preliminarily explained by a dual microbial mechanism: the inhibition of the pro‐inflammatory associated *[Eubacterium]‐oxidoreducens‐group* alongside the expansion of taxa linked to a neuroprotective SCFA metabolic network. As one of the first studies to map these multi‐dimensional “constituent–microbiota–neuroinflammation” interactions for IPCO, our findings highlight its complex, dose‐sensitive bioactivity. Importantly, they underscore the critical need for subsequent pharmacokinetic and direct target engagement studies.

## Graphical abstract

Step 1: Component identification and target screening: The chemical composition of IPCO was characterized using GC–MS, identifying two major fatty acids and six fat‐soluble active substances. A total of 149 potential drug targets were predicted via Pharm Mapper and SwissTargetPrediction. Concurrently, 2029 genes associated with Alzheimer's Disease (AD) were retrieved from the OMIM, TTD, and GeneCards databases. Through the intersection analysis of “ingredient‐target‐disease,” combined with STRING‐based Protein–Protein Interaction (PPI) analysis, Gene Ontology (GO), and KEGG pathway enrichment, the molecular mechanisms underlying the neuroprotective effects of IPCO were elucidated. Step 2: Structural validation (molecular docking): Molecular docking simulations were performed to evaluate the binding affinity between active ingredients and core targets. The results demonstrated that the core component, β‐amyrin, exhibited high binding affinity with key protein targets. Specifically, the binding energies of β‐amyrin with PPARG and ERBB2 were −8.80 and −9.10 kcal/mol, respectively. These high‐affinity interactions provide structural evidence validating the predictions made through network pharmacology. Step 3: In vivo verification (gut microbiota and biochemical markers): In vivo experiments confirmed that IPCO intervention significantly modulated the gut microbiota in AD rats, notably increasing the abundance of the *[Eubacterium]‐oxidoreducens‐group*. Biochemically, IPCO treatment markedly enhanced the levels of SCFAs and the anti‐inflammatory cytokine IL‐10. Furthermore, a significant reduction in hippocampal Al accumulation and inhibition of AChE activity were observed. These findings suggest that IPCO exerts its anti‐AD effects potentially by modulating the gut‐microbiota‐brain axis and improving cholinergic system function. This study systematically elucidated the neuroprotective mechanisms of IPCO against AD through an integrated approach of network pharmacology, molecular docking, and in vivo experiments. Initially, eight core active ingredients were identified in IPCO via GC–MS, with 149 potential targets and 2029 ad‐related genes mapped through multi‐database screening. Molecular docking further validated that the core component, β‐amyrin, exhibited high binding affinities for key targets including PPARG (−8.80 kcal/mol) and ERBB2 (−9.10 kcal/mol). Finally, in vivo verification demonstrated that IPCO intervention significantly modulated the gut‐microbiota‐brain axis by increasing the abundance of the *[Eubacterium]‐oxidoreducens‐group*, elevating levels of SCFAs and IL‐10, while concurrently reducing hippocampal Al accumulation and inhibiting AChE activity.

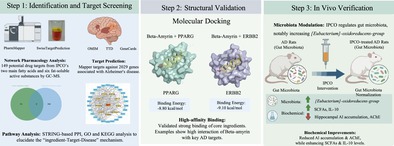

## 1 Introduction

Dementia is a cluster of neurological disorders characterized by progressive cognitive decline, clinically manifested as memory impairment, multidimensional cognitive deficits (including executive function, language, and visuospatial skills), and behavioral abnormalities, ultimately leading to complete loss of independent living capacity in advanced stages. As the dominant subtype, Alzheimer's disease (AD) accounts for 60%–70% of globally confirmed dementia cases, underscoring its core position within the disease spectrum (Monica Moore et al. 2021). Epidemiological data from the World Health Organization reveals that the global dementia population reached 47 million cases (approximately 5% of the elderly population) in 2015, with projections indicating geometric progression: surpassing 75 million by 2030 and escalating to 132 million by 2050, amounting to a new case every 3 s and 9.9 million cases each year (World Health, O 2017). Heavy metal contamination has become a major focus among environmental risk factors because of its neurotoxic effects, ability to accumulate in organisms, and long‐lasting presence in the environment (Raj et al. 2021). Particularly noteworthy is aluminum exposure—despite lacking recognized physiological functions in humans, its chronic accumulation through drinking water, the food chain, and airborne dust has been implicated in triggering neurodegenerative pathologies (Kumar and Gill 2014). Research indicates that environmental factors may influence the onset and progression of AD, with human exposure to aluminum being a significant factor, as aluminum has been detected in the brain tissue of individuals with sporadic AD. The first measurement of aluminum content in the brain tissue of 12 donors diagnosed with familial AD revealed extremely high levels of aluminum (Mirza et al. 2017).

In recent years, with in‐depth research on AD, multiple mechanisms influencing the condition have been proposed. While Aβ plaques and abnormal tau protein phosphorylation are central to AD pathology, several other factors contribute to its development and progression. These include disruptions in acetylcholine signaling, neuroinflammation, oxidative stress, imbalances in biological metal regulation, glutamate system dysfunction, insulin resistance, irregularities in the gut microbiome, disturbed cholesterol balance, and mitochondrial dysfunction (Zhang et al. 2024, 2021; Rostagno 2023; Ju and Tam 2022; Tang et al. 2022; Bai et al. 2022; Bayraktar et al. 2021; Chen, Fan, et al. 2023; Chandra et al. 2023; Shabbir et al. 2021). Pathological mechanism studies reveal that insoluble aluminum salt complexes formed by aluminum ions in brain tissue can deposit in neurons and glial cells, ultimately leading to neuronal degeneration and synaptic dysfunction through multiple pathways such as disrupting microtubule structure, inducing abnormal folding of β‐amyloid proteins, and triggering excessive phosphorylation of tau proteins (Exley 2005; Kaur et al. 2006). SCFAs, gut microbiota metabolites identified in recent studies as potentially neuroprotective, appear to counteract neuronal oxidative stress by augmenting the glutamate‐glutamine shuttle mechanism in cells. Furthermore, studies demonstrate that SCFA supplementation can modulate gut microbiota homeostasis, reduce Aβ deposition, and mitigate tau hyperphosphorylation (Sun et al. 2023). Thus, tracking gut microbiota structure (especially SCFA‐producer abundance) and SCFA concentrations offers a valuable approach in AD research to determine if interventions modify disease course.

AD, a neurodegenerative disorder with a preclinical phase spanning decade, initiates pathological processes involving irreversible molecular, cellular, and neural network damage long before clinical symptoms emerge. Current clinical strategies prioritize delaying disease progression and improving quality of life, yet no curative treatments exist (Atri 2019). Among the FDA‐approved drugs for AD treatment, Aducanumab, Donanemab, and Lecanemab specifically target and effectively clear β‐amyloid (Aβ) plaques, while others like Donepezil and Galantamine alleviate symptoms via cholinergic activation or NMDA receptor antagonism, albeit with limited efficacy and adverse effect risks (Pardo‐Moreno et al. 2022; Buckley and Salpeter 2015; Thompson et al. 2004; Lampela et al. 2017; Campos et al. 2016; Birks and Harvey 2018; Porsteinsson et al. 2008; Shcherbinin et al. 2022; Bobbins et al. 2025; Larkin 2023; Pokhrel et al. 2025). This therapeutic gap has spurred interest in natural neuroprotective agents. Increasingly valued for their health benefits, culinary versatility, and essential fatty acid content, plant seed oils show promise in modulating AD (Rahim et al. 2023). In aluminum‐induced AD models, virgin coconut oil reduces Aβ and hyperphosphorylated Tau levels, mitigates oxidative stress, and enhances neurotransmitter performance (Demirel et al. 2024). Sesame oil, conversely, improves cognitive deficits by suppressing p38 MAPK activation, maintaining BDNF levels, modulating PPAR‐γ/NF‐κB signaling, regulating AChE/Aβ expression, and reducing cerebral oxidative damage (Mohamed et al. 2021). These findings collectively advance experimental support for natural dietary oils as innovative AD intervention strategies.

*Idesia polycarpa* Maxim. (belonging to the Salicaceae family) is a fast‐growing, deciduous tree species widely distributed in subtropical to temperate regions. Its fruit is considered a promising oil resource, with a fruit pulp oil content of about 28% and linoleic acid content ranging from 66% to 81%, which outperforms several traditional vegetable oils in terms of fatty acid composition (Li et al. 2019; Fan et al. 2019). Previous studies have shown that its crude oil is rich in polyphenols and unsaturated fatty acids, demonstrating significant antioxidant and anti‐inflammatory activities in experimental models, mainly attributed to its free radical scavenging ability (Zhang et al. 2023; Guo et al. 2023). However, whether these biological effects can influence the pathological process of AD through the regulation of oxidative stress and neuroinflammatory pathways remains a question with a lack of direct and systematic experimental evidence. Moreover, there is limited information in existing literature regarding the specific composition and quantitative data of its polyphenols and other lipophilic active components. To address this, the present study focuses on the two most abundant fatty acids and six lipophilic active components in IPCO. Using GC–MS, their relative chemical composition is characterized. In combination with network pharmacology and molecular docking methods, potential target sites and molecular mechanisms of action in AD are explored. On this basis, the study further integrates gut microbiota structural analysis and SCFAs metabolic profiling to construct an exploratory, multi‐dimensional, and multi‐omics research framework to reveal the potential associations between IPCO intervention and improvements in pathological markers, neuroinflammation levels, and behavioral performance in AD animal models. This integrated strategy, without performing absolute quantification on individual components, combines chemical composition analysis with mechanistic inference, providing a reasonable theoretical basis for explaining the dose‐dependent neuroprotective effects observed in vivo. This study contributes both theoretically and practically: it supports expanding the functional scope of IPCO components and provides a scientific basis for creating neuroprotective edible oils, thereby boosting the industrial value‐added prospects of *Idesia polycarpa*.

## 2 Materials and Methods

### 2.1 Materials

*Idesia polycarpa* Maxim. was obtained from Jianshi County, Enshi City, Hubei Province, China. The aluminum ion standard solution and magnesium nitrate matrix modifier were sourced from the National Research Center for Nonferrous Metals and Electronic Materials Analysis (Beijing, China) and FULIN SHIJI (Shenzhen, China), respectively. Diethyl ether (Titan, Shanghai) and phosphoric acid (Sinopharm, Shanghai) were acquired from Chinese suppliers. SCFAs—enumerated as acetic, propionic, butyric, isobutyric, valeric, isovaleric, hexanoic, and isocaproic acids (where isocaproic acid functioned as the 4‐methylvaleric acid internal standard)—came from Sigma (USA). Aluminum chloride (AlCl_3_, pale yellow solid) and supplementary reagents originated from Aladdin (Shanghai).

### 2.2 IPCO Extraction

After discarding defective specimens, fresh *Idesia polycarpa* fruits underwent drying at 55°C in a Boxun Medical Biological Instrument Corp (Shanghai) heat pump oven to constant mass. Crude oil extracted via mechanical pressing was subsequently purified by centrifugation (5000 rpm, 20 min) using Heal Force Bio‐Meditech Holdings LTD (Shanghai) equipment.

### 2.3 Determination of IPCO Fatty Acid Composition and Fat‐Soluble Active Substances

Gas chromatography–mass spectrometry (GC–MS) was employed to examine the fatty acid composition and lipid‐soluble bioactive components of the oil, with chromatographic peaks integrated via normalization to determine percentage contents. Following published protocols (Chen, Chen, et al. 2023; Jian et al. 2022), 100 μL of oil underwent methyl esterification: dissolution in 2 mL of n‐hexane, addition of 1 mL of 1 mol/L KOH‐methanol solution, vigorous shaking, and reaction at 60°C for 40 min. After cooling, 3 mL of saturated NaCl solution was added, homogenized, and centrifuged at 3500 r/min for 10 min. The n‐hexane layer was collected, filtered through a 0.45 μm organic membrane, and analyzed via GC under the following conditions: separation was performed on an Agilent 123–5533 dB‐5MS capillary column (30 m × 0.32 mm × 1 μm film thickness; Agilent Technologies, USA) with helium carrier gas at 1.0 mL/min. The injection port temperature was kept constant at 250°C. For the oven program: initial equilibration at 80°C (3‐min isothermal period), followed by an 8°C/min ramp to 150°C (7‐min stabilization), then elevation at 10°C/min to a final 240°C (10‐min terminal hold). Full‐scan mass spectral data (*m*/*z* 50–600) were recorded via electron impact ionization (70 eV) employing the integrated GC–MS platform (Peng et al. 2013).

### 2.4 Network Pharmacology

#### 2.4.1 Prediction of Active Substances and Targets in IPCO

GC–MS characterization identified fatty acids and lipid‐soluble bioactive compounds in IPCO, with chemical nomenclature sourced from PubChem (https://pubchem.ncbi.nlm.nih.gov/) and associated 2D structural data retrieved as SDF files. Using reverse pharmacophore matching via the PharmMapper platform (http://www.lilab‐ecust.cn/), potential human protein targets of representative blood‐absorbed components were predicted, with thresholds of norm fit > 0.8. SMILES‐formatted data were imported into the Swiss Target Prediction database (http://www.swisstargetprediction.ch/) (Tang et al. 2023), restricted to “ *Homo sapiens* ”, and component‐associated targets were collected. Targets were filtered by probability > 0, duplicates removed, yielding definitive IPCO‐targeted proteins.

#### 2.4.2 AD Target Screening and Intersecting Target Acquisition

We sourced AD targets from three databases: OMIM (https://omim.org/) (Hamosh et al. 2002), GeneCards (https://www.genecards.org/) (Safran et al. 2010), and TTD (https://www.idrblab.net/) (Zhou et al. 2022). Post‐deduplication, target names were formally corrected to standardized nomenclature using UniProt (https://www.uniprot.org/) (UniProt Consortium 2018). The data in the GeneCards database were filtered by a “relevance score > 20”. Duplicates were eliminated from the three databases, and the target protein of AD disease was obtained. The WeiShengXin platform (https://www.bioinformatics.com.cn/) was used to visualize the intersection targets of IPCO and AD disease.

#### 2.4.3 Protein Interaction (PPI) Network Construction and Analysis

Shared targets were imported into the STRING database (https://cn.string‐db.org/) (Szklarczyk et al. 2019), with species limited to *Homo sapiens* and a minimum interaction score (confidence > 0.400) set to construct a protein–protein interaction (PPI) prediction network for IPCO's action on AD. The PPI network was downloaded as a TSV file, imported into Cytoscape 3.9.1, and visualized. Core targets were identified using the CytoNCA plugin by filtering nodes with topological parameter degree values > median (13.5) (Shannon et al. 2003).

#### 2.4.4 GO and KEGG Enrichment Analysis

The obtained intersecting targets were imported into the DAVID database (https://David.ncifcrf.gov/), and the GO biological function and KEGG signaling pathway enrichment analysis were performed, and the species was selected as “ *Homo sapiens* ”, and the results were visualized by the online mapping website of the WeiShengXin. Bar or bubble charts were used to depict the top 10 biological processes (BP), cellular components (CC), and molecular functions (MF), as well as the top 20 KEGG pathways, selected according to *p*‐value ranking.

### 2.5 Molecular Docking and Docking Protocol Validation

In this study, based on the IPCO‐active substance‐target‐AD network, three core active ingredients with a Degree ≥ 17 and key targets with a Degree > 13.5 were screened to construct a molecular docking system. First, the SDF structures of the small molecules and the crystal structures of the target proteins were obtained from the PubChem and PDB databases, respectively. Subsequently, PyMOL 3.11 was used to remove water molecules and the original ligands from the target proteins to expose the active sites, and the conformational energy of the small molecules was optimized using Chem3D 23.1.1. Next, AutoDockTools 1.5.7 was employed to prepare the receptor (by adding hydrogen atoms and assigning Gasteiger charges) and the ligands (by setting rotatable bonds), generating PDBQT files. Semi‐flexible ligand molecular docking was then performed using AutoDock Vina 1.2.3. During docking, the grid box was centered on a known active pocket, covering the entire binding region, with its dimensions defined by the crystallographic ligand's binding coordinates, and the exhaustiveness was set to 10 to optimize the conformational search. To validate the effectiveness of the docking parameters, a re‐docking experiment was conducted: the native ligands were extracted from the cocrystal structures of the target proteins (PDB ID: 2F4B‐EHA; AKT1‐4IP; 2GIU‐FBR), removed, and then re‐docked using the same parameters. The calculated root mean square deviation (RMSD) values of the obtained conformations from the original crystal conformations were 2.007 Å, 0.902 Å, and 1.393 Å, respectively, indicating that the docking protocol is reliable.

### 2.6 MM‐GBSA Combined Free Energy Calculation

To obtain a more accurate estimation of binding affinity, MM‐GBSA calculation was further performed to analyze the binding free energy of the top‐scored complex conformation obtained from docking in Section 2.5, and the resulting MM‐GBSA binding energy was ultimately adopted as the primary evaluation metric. Specifically, the complex was saved in PDB format and imported into the Schrodinger Suite 2023 platform. The complex was first systematically optimized using the Protein Preparation Wizard module, including hydrogen addition, optimization of the hydrogen‐bond network at pH 7.4, and energy minimization. Subsequently, the MM‐GBSA calculation was carried out with the Prime module under the VSGB solvation model. In addition, non‐covalent interactions in the binding mode were analyzed and visualized using the Interaction Diagram tool within the same software platform.

### 2.7 In Vivo Validation

#### 2.7.1 Animals and Design

Male Wistar rats aged 6 weeks were acquired from the Hubei Provincial Laboratory Animal Research Center (Quality Certification Number: SCXK (E) 2020‐0018) and placed in the Specific Pathogen Free (SPF) Barrier Laboratory (Facility License Number: SYXK (E) 2023‐0138) of the Animal Experiment Center of Enshi Prefecture Central Hospital. Rats were allowed to eat freely and were placed in a controlled environment with a 12‐h light–dark cycle, 65% ± 5% humidity, and 22°C ± 2°C temperature. All animal procedures conducted in this investigation received ethical approval (Approval No. 202408003) from the Institutional Animal Care and Use Committee (IACUC) of Enshi Prefecture Central Hospital's Animal Experiment Center. All rats are given a week to acclimatize to access to standard pellet feed and water.

#### 2.7.2 Sample Size Estimation

An a priori power analysis was conducted to determine the required sample size based on the primary outcome measure: aluminum content in the hippocampus. According to previously reported data by Liu et al. (2024a), the hippocampal aluminum content in the model group (17.65 ± 2.52 μg/g) and the treatment group (8.96 ± 1.29 μg/g) yielded a mean difference of 8.69 μg/g and a pooled standard deviation of 2.00 μg/g. This resulted in a Cohen's d effect size assumption of 4.34. Using the SPSSAU online module with a two‐sided Type I error rate (*α*) of 0.05 and a statistical power (1 − *β*) of 80%, the analysis indicated that a minimum of 3 rats per group was required. For other indicators where specific pilot data were unavailable, sample sizes were determined based on established methodologies in previous literature (Chen, Weng, et al. 2022). Comprehensive details regarding the effect size assumptions for all major outcomes are provided in Table S1. In the Results section, we report exact *p*‐values and partial *η* ^2^ for all primary comparisons to ensure statistical transparency.

#### 2.7.3 Randomization and Blinding

Randomization was implemented through simple randomization, and the sequence of random numbers was produced using R software (version 4.3.1). This process of randomization was executed by statisticians who were not participants in the study. CWJ performs experimental operations, including gavage, changing feed bedding, etc.; CYB did not know the grouping in advance, and then carried out the Morris water maze test, the determination of aluminum ions in the hippocampus, etc. All data obtained were analyzed by variance by HXF. Subsequently, CWJ performed visual analysis, but the grouping was not known, and finally LK organized the data and visualization images.

#### 2.7.4 Experiment Design

The dosage regimen of AlCl_3_ (100 mg/kg body weight/day, via oral gavage, for 48 consecutive days) employed in this study was designed to mimic a chronic, low‐level aluminum exposure state associated with the development of AD pathology, rather than to induce acute neurotoxicity. The selection of this specific dose and duration was based on a protocol widely adopted in prior AD model research, which has been shown to stably induce AD‐related cognitive deficits and neuropathological alterations (Firdaus et al. 2022). Based on this rationale, the experimental design (including the cycle, grouping, aluminum chloride concentration, and IPCO dosage) and sample size were determined with reference to previous similar studies (Weng et al. 2020; Gupta et al. 2023; Ghaderi et al. 2022).

Rats were randomly divided into five groups (*n* = 8): the Control group received an equivalent volume of saline daily via gavage, with standardized husbandry procedures implemented to match experimental conditions; the Model group received 100 mg/kg body weight AlCl_3_ daily by gavage (Weng et al. 2020); the Low‐, Medium‐, and High‐dose IPCO groups received the same AlCl_3_ dose combined with 1.0, 2.0, and 3.0 mL/kg body weight IPCO, respectively. All treatments lasted for 48 consecutive days. To strictly prevent unintended aluminum exposure in control animals, operational procedures were optimized: all feed came from the same batch and was provided using dedicated, cleaned tools in a fixed sequence from control to treatment cages; aluminum treatment (preparation of dosed drinking water) was conducted in a separate fume hood or dedicated area, with utensils strictly segregated and washed separately from those used for controls; animals were housed in separate cages, and all supplies for treated groups were clearly labeled and stored separately. The maintenance feed was supplied by Liaoning Changsheng Bioengineering Co. Ltd. (SCXK (Liao) 2020‐0002), with its composition shown in Table S2.

The mice used in this study were of the same strain, age, and sex as the experimental animals and were housed under strictly controlled specific pathogen‐free (SPF) conditions. To ensure consistency of the experimental results, the baseline characteristics of animals in each group were aligned as closely as possible during group allocation. For biochemical analyses, three out of six animals that completed the behavioral tests were randomly selected for the determination of aluminum ion content, oxidative stress markers, inflammatory cytokines, and acetylcholinesterase activity. All data are presented as scatter bar charts, allowing each data point (*n* = 3) to be clearly displayed, which helps to reveal variability inherent in small sample sizes. To ensure consistency of the experimental results, the baseline characteristics of animals in each group were aligned as closely as possible during group allocation. For biochemical analyses, three out of six animals that completed the behavioral tests were randomly selected for the determination of aluminum ion content, oxidative stress markers, inflammatory cytokines, and acetylcholinesterase activity. All data are presented as scatter bar charts, allowing each data point (*n* = 3) to be clearly displayed, which helps to reveal variability inherent in small sample sizes.

After a 5‐day washout period, behavioral tests were conducted over 7 days. On day 60, rats were anesthetized with tribromoethanol and sacrificed to collect brain tissue and stool samples for further analysis.

#### 2.7.5 Morris Water Maze (MWM) Test

The methods of Vorhees and Williams (2006) and Liu et al. (2024b) were used to modify the Morris water maze experiment and it was evaluated 5 days after the gavage was stopped. The experiment was divided into two parts: (1) positioning navigation training: 4 times a day for 4 consecutive days, each time the rat was put into the water from the face wall of the unified starting point, and the incubation period of the stage within 90 s was recorded (90 s for those who were unsuccessful and guided to the platform for 10 s), and the data on the 5th day was the final result; (2) Spatial exploration test: 24 h post‐training, the platform was removed. Rats entered the pool from a fixed starting point, and crossings of the former platform location within 120 s were recorded to assess spatial memory.

#### 2.7.6 Determination of Aluminum Content in Hippocampal Tissues of Rats in Each Group

Sun et al. (Sun and Chen 2007) enhanced the detection of aluminum in hippocampal tissue using graphite furnace atomic absorption spectrometry. 20 mg of the sample was accurately weighed, digested with nitric acid and then reduced to 10 mL (1% nitric acid medium), and the standard curve was established with a gradient of 0–100 μg/L aluminum standard solution. The instrument operated at 309.3 nm wavelength with 15 mA lamp current and 0.7 nm slit width, featuring automatic co‐injection of 20 μL sample and 5 μL matrix modifier. Zeeman background correction and argon protection system, quantitative analysis with peak area, and simultaneous blank control to ensure the accuracy of detection.

#### 2.7.7 Assessment of Antioxidant Activity in the Hippocampus

Hippocampal tissues (≈0.05 g) were homogenized on ice in kit‐provided extraction buffer, centrifuged (10,000 rpm, 10 min, 4°C), and supernatants were analyzed. Malondialdehyde (MDA), glutathione peroxidase (GSH‐Px), and superoxide dismutase (SOD) were measured using commercial kits (Suzhou Michy Biotechnology Co. Ltd.; Cat# M0106B, M0304B, M0102B) on an Infinite M200 Pro microplate reader (TECAN). Each sample was run in triplicate; intra‐assay coefficients of variation (CV) were 0.77%–1.92% (SOD), 1.53%–3.76% (GSH‐Px), 1.70%–5.36% (MDA). Spike‐recovery rates for SOD, MDA, and GSH‐Px were 102.3%, 101.8%, and 104.0%, respectively. Methodologically, SOD and GSH‐Px activities were derived from theoretical formulas, and MDA from a pre‐validated curve (*y* = 0.0391*x*−0.0076, *R* ^2^ = 0.9997). All assays were performed blinded, with samples randomized and identified only by numerical codes.

#### 2.7.8 Hippocampal Inflammatory Factor Determination

Hippocampal tissue (0.05 g) was homogenized with 450 μL physiological saline at 60 Hz (4 × 60 s) under −20°C, and the supernatant was collected post‐centrifugation. Levels of IL‐4, IL‐6, IL‐10, IL‐1β, and TNF‐α in serum and tissue were quantified using rat‐specific ELISA kits (DUMABIO, Shanghai; Cat. No. DM‐D8469; DM‐D8470; DM‐D8471; DM‐D8467; DM‐D8473) and a TECAN Infinite M200 Pro reader at 450 nm, following a randomized and blinded protocol. The assay demonstrated excellent linearity (*R* ^2^) and recovery rates for IL‐4 (*R* ^2^ = 0.99981, 103.1%), IL‐6 (*R* ^2^ = 0.99997, 101.2%), IL‐10 (*R* ^2^ = 0.99904, 102.3%), IL‐1β (*R* ^2^ = 0.99994, 102.1%), and TNF‐α (*R* ^2^ = 0.99969, 101.6%). Intra‐group CV ranged from 0.19% to 1.66% (all < 2%), confirming high assay precision and reproducibility. To minimize bias, a blinded experimental design was employed; samples were processed in a randomized order and identified only by numerical codes, ensuring that operators remained unaware of the experimental conditions during sample loading, incubation, and plate reading.

#### 2.7.9 Hippocampal Acetylcholinease Activity Assay

Approximately 0.05 g of hippocampal tissue (±0.001 g) was weighed into a 2 mL grinding tube with grinding beads, mixed with 450 μL of physiological saline, and homogenized at 60 Hz for 60 s under −20°C. The grinding cycle was repeated four times, and the homogenate was centrifuged at 5000 rpm for 10 min. The supernatant was kept on ice throughout the process. One aliquot was used to determine protein concentration using a BCA assay kit (Jiancheng Co. Ltd., Nanjing, China), and another was analyzed with an ELISA kit. Samples were processed randomly, identified by numerical codes, and operators were blinded to the experimental conditions. Acetylcholinesterase (AChE) activity was measured in triplicate using a kit from Nanjing Jiancheng Bioengineering Institute (Cat# A024‐1‐1), with intra‐assay CVs ranging from 0.89% to 2.46%.

#### 2.7.10 Fecal DNA Extraction, PCR Amplification, Sequencing, and Data Analysis

Fecal sequencing analysis was conducted by Wekemo Tech Group Co. Ltd. (Shenzhen, China). Genomic DNA was isolated using a CTAB‐based kit (Nobleryder, Beijing) and assessed through 1% agarose gel electrophoresis. Amplification of 16S rRNA V3‐V4 regions with primers 341F/806R preceded purification and Illumina NovaSeq 6000 sequencing of amplicons. Raw data were processed in QIIME2 (v1.22.0) using the DADA2 plugin for quality filtering, denoising, and chimera removal, generating an ASV table (Callahan et al. 2016). Taxonomic annotation was performed against the SILVA database (v138, 97% similarity) with contaminant removal. Alpha diversity (ASVs, Chao1, Shannon/Simpson indices) and beta diversity (Bray‐Curtis distance) were calculated via the Wekemo Bioincloud platform (https://www.bioincloud.tech) (Gao et al. 2024), visualized through PCoA/NMDS, and intergroup microbial community differences were analyzed using PLS‐DA. Spearman correlation analysis combined with Pheatmap (v1.0.12) was employed to generate heatmaps illustrating associations between microbial taxa and metabolic parameters.

#### 2.7.11 Determination of SCFAs in Feces

Quantification of fecal SCFAs was conducted by Wekemo Tech Group (Shenzhen, China). Calibration standards were generated through serial dilution of aqueous stock solutions (100 mg/mL) containing acetic, propionic, butyric, isobutyric, valeric, and isovaleric acids. Hexanoic acid stock (100 mg/mL) was prepared in diethyl ether and similarly diluted. The internal standard (4‐methylvaleric acid) was dissolved in diethyl ether at 375 μg/mL. Calibration standards across 10 concentrations (0.02–500 μg/mL) were prepared by combining 200 μL of six‐acid working standards, 100 μL of 15% phosphoric acid, 20 μL of hexanoic acid standard, 20 μL of internal standard, and 260 μL diethyl ether. Stock solutions were stored at −20°C, with working solutions prepared fresh before analysis.

SCFA extraction (Han et al. 2018) commenced with sample homogenization in 1.5 mL microtubes containing 500 μL deionized H₂O and 100 mg glass beads (0.5 mm diameter; 60 s processing). Subsequent centrifugation (10 min, 4°C, 12,000*g*) yielded supernatants, of which 200 μL aliquots were mixed with 100 μL 15% H_3_PO_4_, 20 μL internal standard (4‐methylvaleric acid, 375 μg/mL), and 280 μL diethyl ether. After vortex mixing (60 s), identical centrifugation parameters were reapplied. The ether phase was subjected to instrumental analysis.

### 2.8 Data Analysis

All data was analyzed and visualized using OriginPro 2024 (Origin Lab Corporation, Massachusetts, USA). Continuous variables are expressed as mean ± standard deviation (SD). Normality was assessed using the Shapiro–Wilk test. For normally distributed data, one‐way ANOVA and Tukey's post hoc test were used to evaluate the differences between groups. The non‐normally distributed data were analyzed by Kruskal–Wallis test and Dunn post hoc correction. The statistical significance was 95% confidence level (*p* < 0.05).

## 3 Result

### 3.1 IPCO Anti‐AD Active Ingredient Discovery Study Based on Network Pharmacology Strategy

**FIGURE 1:**
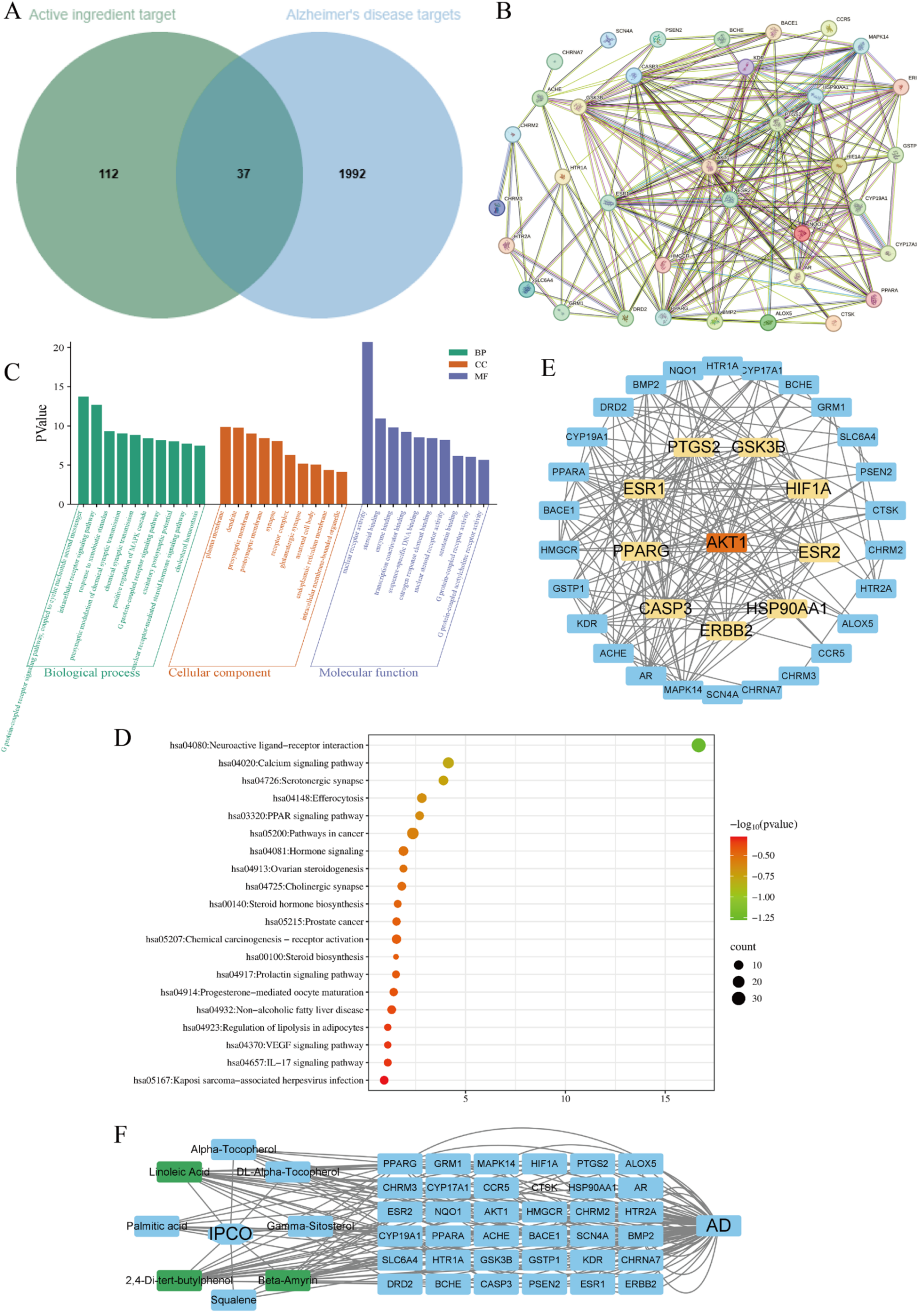
Network pharmacological analysis of IPCO and AD. Vene diagram of the active ingredient‐related targets and AD‐related gene targets of the IPCO (A), The target protein of IPCO interacts with the target protein of AD (B), GO function bar chart of the top 10 potential targets (C), KEGG functional bubble diagram of top 20 potential targets (D). CytoNCA screening core target network diagram (E). IPCO—active ingredient–common target—AD network diagram (F).

Based on GC–MS results (Figure S1), we identified two primary fatty acids—linoleic acid and palmitic acid—along with six bioactive components (Table 1). A total of 149 potential drug targets were predicted using the Swiss Target Prediction platform and PharmMapper database, while 2029 ad‐related genes were integrated from OMIM, GeneCards, and TTD databases. Venn diagram analysis revealed 37 key intersecting targets (Figure 1A). GO/KEGG enrichment via the DAVID database identified 586 significantly enriched pathways: 357 biological processes (BP) including intracellular receptor signaling pathways, presynaptic regulation of chemical synaptic transmission, and positive regulation of MAPK cascade; 47 cellular components (CC) localized to plasma membranes and dendrites; and 131 molecular functions (MF) associated with nuclear receptor activity, steroid binding, and enzyme binding (Figure 1C). KEGG pathway analysis highlighted 51 critical pathways, such as neuroactive ligand‐receptor interactions, calcium signaling, and serotonergic synapses (Figure 1D). The PPI network constructed using STRING (Figure 1B) was topologically analyzed in Cytoscape, yielding 10 core targets with degree values ≥ 14 (Figure 1E; Table 2). These results were integrated to construct a multidimensional network model (“IPCO‐bioactive components‐shared targets‐AD”), which visually represents a hypothesized mechanism involving multi‐component, multi‐target, and multi‐pathway interactions potentially relevant to neuroinflammatory regulation (Figure 1F). Based on their high connectivity within this network model (degree ≥ 17), three candidate core compounds—linoleic acid ((Z,Z)‐9,12‐octadecadienoic acid), Beta‐amyrin, and 2,4‐di‐tert‐butylphenol—were prioritized for further in silico evaluation via molecular docking against selected hub targets.

**TABLE 1:** There are two main fatty acids and fat‐soluble active substances in IPCO.

Number	Component	Molecular formula	Degree	Sample number		
				IPCO 1 (%)	IPCO 2 (%)	IPCO 3 (%)
1	((Z,Z)‐9,12‐octadecadienoic acid)	C_18_H_32_O_2_	17.00	47.88	36.30	31.91
2	Palmitic acid	C_16_H_32_O_2_	9.00	23.82	20.00	21.92
3	2,4‐Di‐tert‐butylphenol	C_14_H_22_O	17.00	0.85	0.91	0.89
4	Squalene	C_30_H_50_	4.00	0.27	0.26	0.35
5	Beta‐Amyrin	C_30_H_50_O	18.00	0.17	0.17	0.14
6	Gamma‐Sitosterol	C_29_H_50_O	8.00	0.27	0.25	0.35
7	DL‐Alpha‐Tocopherol	C_29_H_50_O_2_	8.00	0.10	0.18	0.21
8	Alpha‐Tocopherol	C_29_H_50_O_2_	8.00	/	0.18	0.01

*Note:* In network pharmacology, degree centrality (Degree) directly reflects node importance—higher values denote greater network influence. Additionally, IPCO contains supplementary fatty acids beyond those tabulated, including saturated forms (Myristic through Tetracosanoic acids) and unsaturates (Palmitoleic, Eicosenoic, Oleic, Linolenic acids).

**TABLE 2:** Key targets with a moderate value of ≥ 14 in IPCO.

Target protein	*AKT1*	*CASP3*	*ESR1*	*PTGS2*	*PPARG*	*GSK3B*	*HIF1A*	*ESR2*	*HSP90AA1*	*ERBB2*
Degree	26	23	22	22	22	20	17	16	16	14
UniProt ID	P31749	P42574	P03372	P35354	P37231	P49841	Q16665	Q92731	P07900	P04626

*Note:* The following is the full name of the target protein.

Abbreviations: *AKT1*, RAC‐alpha serine/threonine‐protein kinase; *CASP3*, Caspase‐3; *ERBB2*, receptor tyrosine‐protein kinase erbB‐2; *ESR1*, estrogen receptor; *ESR2*, estrogen receptor beta; *GSK3B*, glycogen synthase kinase‐3 beta; *HIF1A*, hypoxia‐inducible factor 1‐alpha; *HSP90AA1*, heat shock protein HSP 90‐alpha; *PPARG*, peroxisome proliferator‐activated receptor gamma; *PTGS2*, prostaglandin G/H synthase 2.

### 3.2 Activity Validation and Target Confirmation Studies Guided by Molecular Docking

**FIGURE 2:**
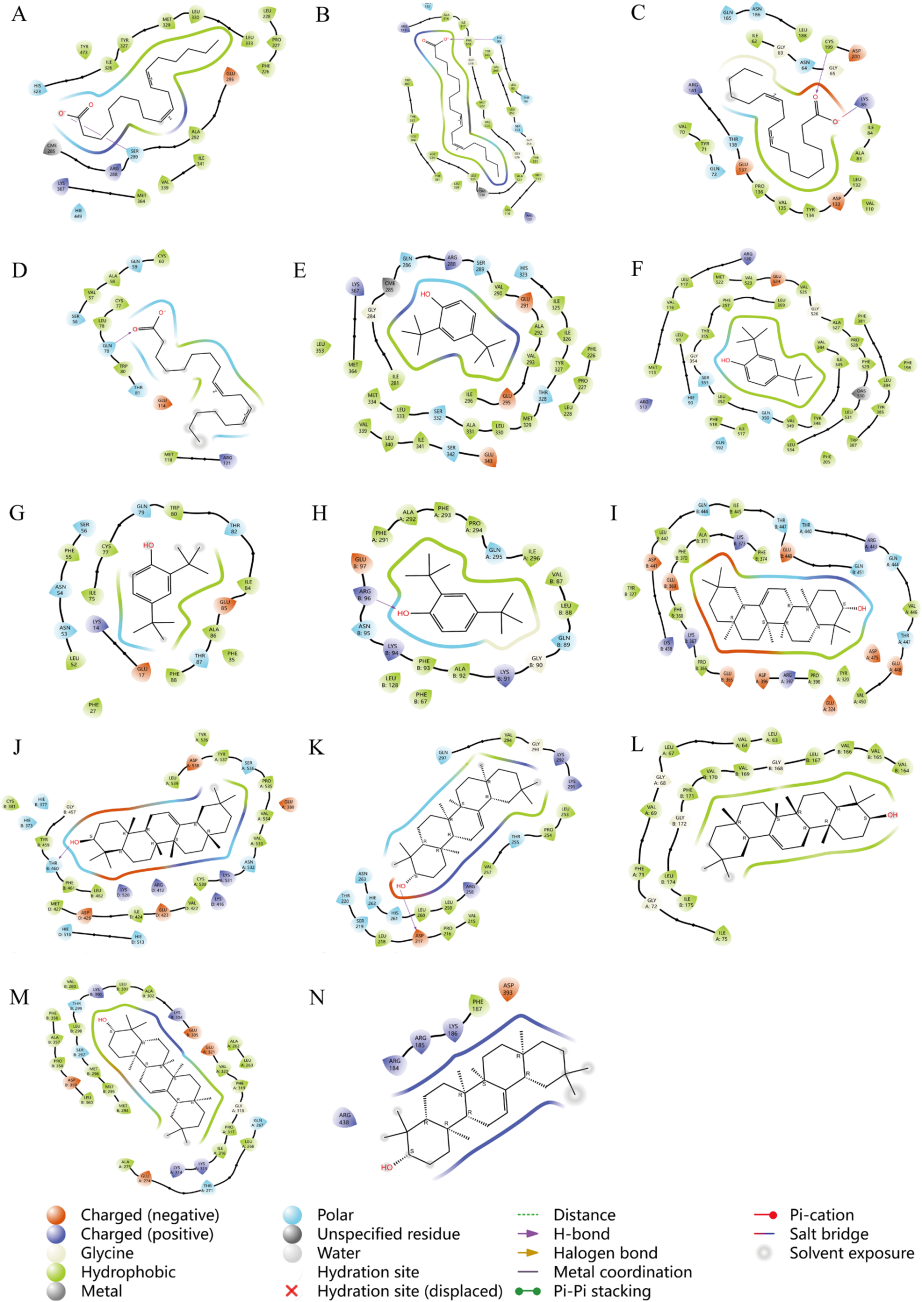
Docking of small molecules with high‐affinity cross‐targets using Schrodinger molecules. Docking diagrams of *PPARG* with linoleic acid (A), *PTGS2* with linoleic acid (B), *AKT1* with linoleic acid (C), *GSK3B* with linoleic acid (D), *PPARG* with 2,4‐di‐*tert*‐butylphenol (E), *AKT1* with 2,4‐di‐*tert*‐butylphenol (F), *GSK3B* with 2,4‐di‐*tert*‐butylphenol (G), *PTGS2* with 2,4‐di‐*tert*‐butylphenol (H), *PPARG* with β‐amyrin (I), *ESR1* with β‐amyrin (J), *HIF1A* with β‐amyrin (K), *ERBB2* with β‐amyrin (L), *PTGS2* with β‐amyrin (M), and *ESR2* with β‐amyrin (N).

To validate the docking results and obtain more rigorous thermodynamic data, a hierarchical computational strategy was implemented, utilizing AutoDock Vina for initial screening followed by MM‐GBSA refinement for representative complexes (Table 3). Compared to the simplified Vina scoring function, MM‐GBSA provides superior reliability by balancing desolvation penalties and hydrophobic interactions through sophisticated molecular mechanics force fields and solvent models, yielding binding free energy estimates that align more closely with physical reality. After ensuring parameter reliability via re‐docking (see Section 2.5), a threshold of < −5 kcal/mol was defined as a significant indicator of ligand‐receptor interaction potential. Our simulations revealed that multiple IPCO components possess high affinity for AD‐related targets, with MM‐GBSA ΔG Bind values significantly below −5 kcal/mol; notably, Linoleic acid exhibited a binding energy of −49.37 kcal/mol with *PPARG*. Structural analysis via Schrödinger's interaction diagrams (Figure 2) showed that Linoleic acid is stabilized within the binding pocket through a key hydrogen bond with SER 289 and hydrophobic contacts with residues such as ILE 326 and TYR 327, which is consistent with the predominant residues identified in energy decomposition. Furthermore, specific hydrogen‐bonding patterns were identified for 2,4‐Di‐tert‐butylphenol with *GSK3B* and Beta‐Amyrin with *ESR1*/*HIF1A*, collectively providing a robust structural and thermodynamic basis for the observed binding affinities.

**TABLE 3:** The binding energy of the core target to its active small molecule.

Active ingredient	Target protein	Binding energy (kcal/mol)	MM‐GBSA ΔG bind
((Z,Z)‐9,12‐octadecadienoic acid)	*PPARG*	−5.70	−49.37^#^
((Z,Z)‐9,12‐octadecadienoic acid)	*PTGS2*	−6.80	−34.09^#^
((Z,Z)‐9,12‐octadecadienoic acid)	*AKT1*	−3.90	−21.17^#^
((Z,Z)‐9,12‐octadecadienoic acid)	*GSK3B*	−4.70	−35.52^#^
2,4‐Di‐tert‐butylphenol	*PPARG*	−7.00	−54.26
2,4‐Di‐tert‐butylphenol	*PTGS2*	−7.40	−47.88
2,4‐Di‐tert‐butylphenol	*AKT1*	−5.30	−29.67
2,4‐Di‐tert‐butylphenol	*GSK3B*	−6.40	−45.10^#^
Beta‐Amyrin	*PPARG*	−8.80	−56.64
Beta‐Amyrin	*ESR1*	−7.90	−49.82^#^
Beta‐Amyrin	*HIF1A*	−7.40	−21.21^#^
Beta‐Amyrin	*ERBB2*	−9.10	−55.64
Beta‐Amyrin	*PTGS2*	−2.70	−43.82
Beta‐Amyrin	*ESR2*	−7.90	−28.52

*Note:* The hash symbol (#) denotes hydrogen bonding between active molecules and target proteins.

### 3.3 IPCO Has the Potential to Improve Memory and Cognitive Impairment in Rats

**FIGURE 3:**
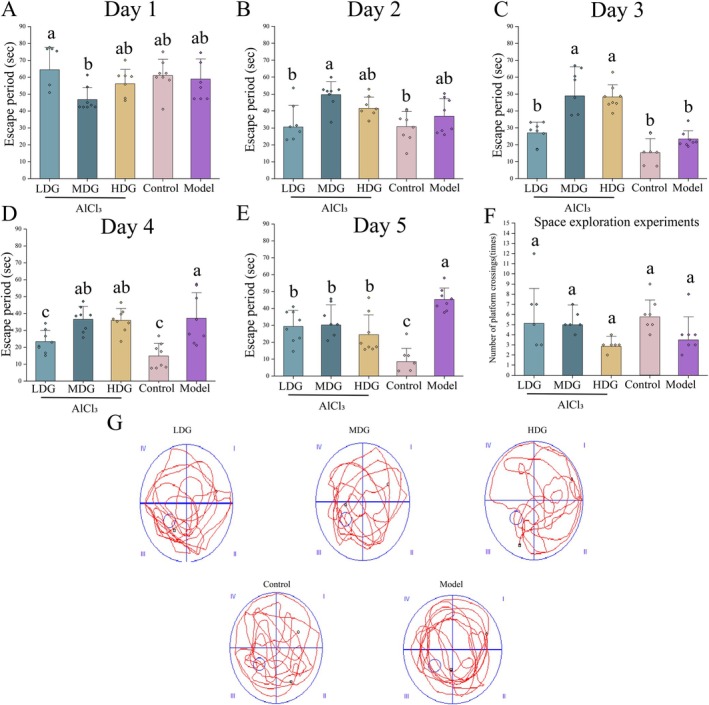
Effect of IPCO on memory and learning ability in aluminum chloride‐treated rats. The reference memory task (4 trials per day for 4 consecutive days); Day 1 (A), Day 2 (B), Day 3 (C), Day 4 (D). Day 5 (E). Space exploration experiments (F). Traces in the probe trial (G). Values are expressed as mean ± SD from independent replicates (*n* = 6). Statistical analysis was performed using one‐way ANOVA with Tukey's post hoc test. Values with different letters are significantly different (*p* < 0.05). The combination of letters (e.g., “ab”) indicates that the value is not significantly different from those marked with either “a” or “b”, with corresponding exact *p*‐values detailed in the manuscript.

AD is characterized by progressive memory impairment and cognitive decline. To assess the intervention effect, we constructed a MWM system with gradient doses (Monica Moore et al. 2021). The aim was to quantify the impact of IPCO doses on spatial learning and memory retention in AD model rats. Results showed a decrease in escape latency during navigation training (days 1–4) across all groups (Figure 3A–D). No significant differences were observed on days 1–2 (Figure 3A,B). On day 3, a one‐way ANOVA revealed a significant treatment effect on latency (*F* (4, 35) = 18.97, *p* < 0.0001, partial *η* ^2^ = 0.684), with the model group showing shorter latency than the medium‐ and high‐dose groups (*p* < 0.0001, *p* = 0.0001), but similar to the control group (Figure 3C). On day 4, the model group showed a significant increase in latency, longer than the control and low‐dose groups (*p* = 0.0003, *p* = 0.03836) (Figure 3D). In the working memory probe test, the model group had the longest latency (45.29 ± 6.77 s), while the low, medium, and high‐dose groups had latencies of 29.44 ± 9.50, 30.26 ± 11.90, and 24.53 ± 11.67 s, respectively, all longer than the control group (8.39 ± 7.95 s) (Figure 3E). Although the number of platform crossings was not significant, both the high‐dose (2.88 ± 0.99 times) and model (3.50 ± 2.27 times) groups had the lowest values (Figure 3F). In summary, chronic AlCl_3_ exposure induced AD‐like phenotypes in rats, including impaired spatial memory.

### 3.4 IPCO Reduces the Concentration of Aluminum in the Hippocampus

**FIGURE 4:**
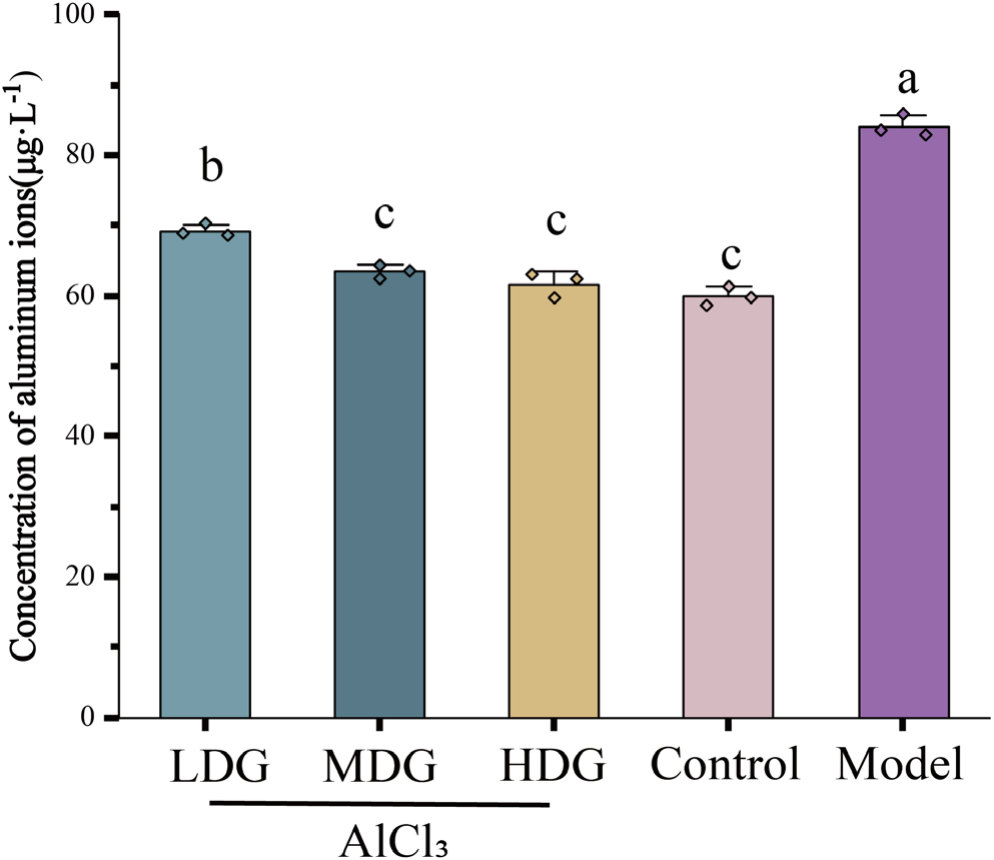
Effect of IPCO on aluminum chloride‐induced Alzheimer's disease in rats. Values are expressed as mean ± SD from independent replicates (*n* = 3). Statistical analysis was performed using one‐way ANOVA with Tukey's post hoc test. Values with different letters are significantly different (*p* < 0.05). The combination of letters (e.g., “ab”) indicates that the value is not significantly different from those marked with either “a” or “b”, with corresponding exact *p*‐values detailed in the manuscript.

To ascertain whether dietary aluminum could have influenced the experimental outcomes, we first measured baseline aluminum concentrations in the diet at the beginning of the study, which were found to be 75.72 ± 7.56 μg/L (Table S3). Previous reports (Martinez et al. 2017; Walton 2009) suggest that dietary aluminum may contribute to elevated baseline levels. Hippocampal aluminum levels were quantified using graphite furnace atomic absorption spectrometry (GFAAS). A one‐way ANOVA revealed an extremely significant effect of treatment on hippocampal aluminum accumulation (*F* (4, 10) = 158.59, *p* < 0.0001, partial *η* ^2^ = 0.985). The results demonstrated that the Model group had significantly higher aluminum concentrations (84.11 ± 1.57 μg/L) compared to all IPCO‐treated groups (low dose: 69.28 ± 0.92 μg/L; medium dose: 63.44 ± 0.96 μg/L; high dose: 61.71 ± 1.76 μg/L; *p* < 0.0001). As anticipated, the Control group also exhibited elevated aluminum levels (59.91 ± 1.35 μg/L; *p* < 0.0001), which raised the overall baseline across groups, yet remained significantly lower than those in the Model group (Figure 4). This analysis was based on five experimental groups with a total sample size of *N* = 15. These findings imply that while dietary aluminum contributed to a generally elevated baseline, it was not the primary cause of the markedly higher aluminum levels in the Model group. This study provides critical evidence for further investigation into the neuroprotective mechanisms of IPCO and its role in regulating aluminum metabolism. In summary, chronic AlCl_3_ exposure successfully induced a series of phenotypes consistent with core AD features in Whistar rats, including increased aluminum ion concentration in the hippocampus.

### 3.5 Evaluation of Hippocampal Antioxidant Activity, Inflammatory Factors and AChE Concentration

**FIGURE 5:**
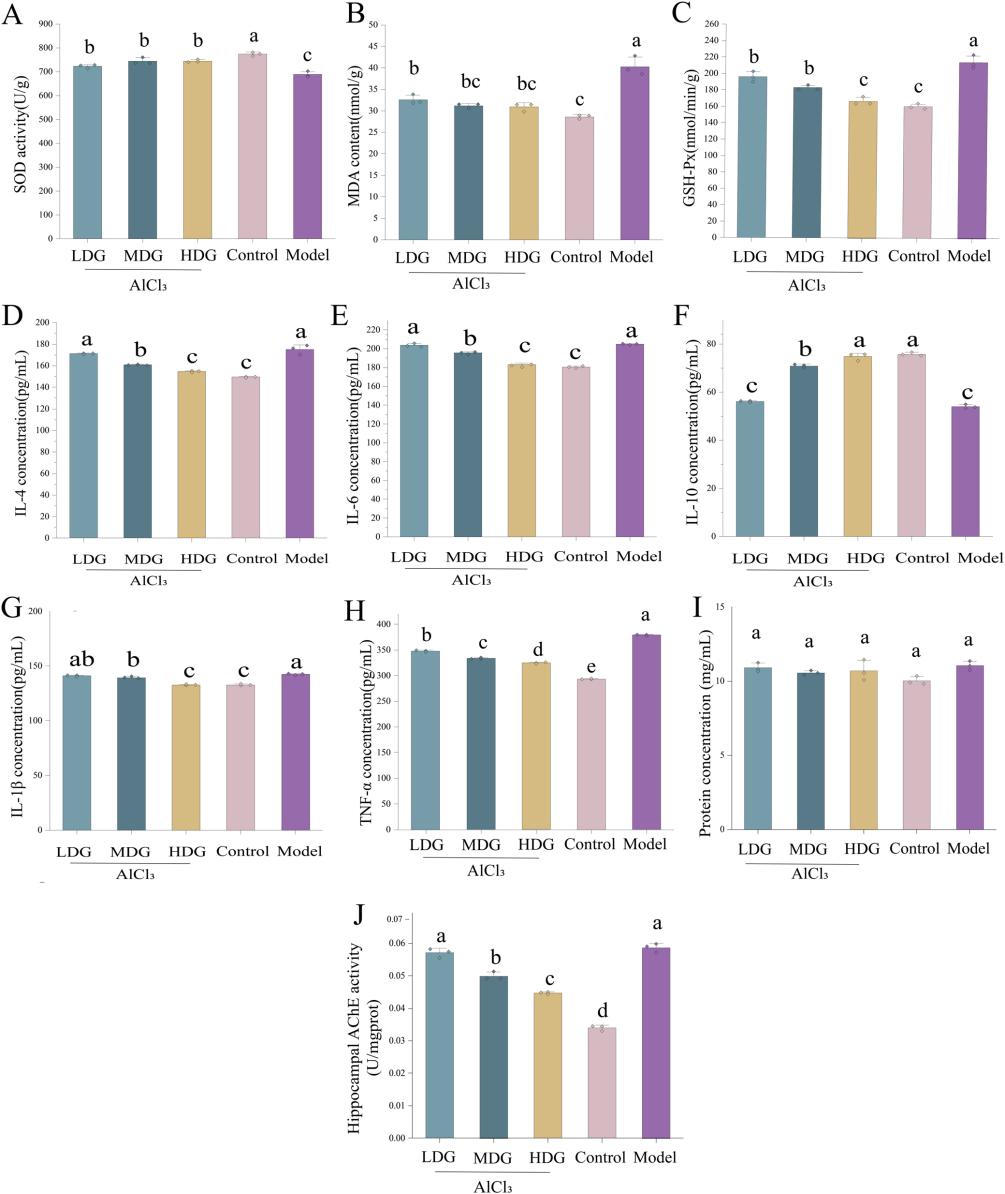
Effects of IPCO on antioxidant activity, inflammatory factors, and AChE concentration in aluminum chloride‐induced Alzheimer's disease. SOD activity (A), MDA content (B), GSH‐Px (C), IL‐4 content (D), IL‐6 content (E), IL‐10 content (F), IL‐1β content (G), TNF‐α content (H). Hippocampus protein concentration (I), AChE concentration in the hippocampus (J). Values are expressed as mean ± SD from independent replicates (*n* = 3). Statistical analysis was performed using one‐way ANOVA with Tukey's post hoc test. Values with different letters are significantly different (*p* < 0.05). The combination of letters (e.g., “ab”) indicates that the value is not significantly different from those marked with either “a” or “b”, with corresponding exact *p*‐values detailed in the manuscript.

This study examined the effects of different IPCO doses on oxidative stress (SOD, GSH‐Px, MDA) and neuroinflammatory cytokines (IL‐6, IL‐1β, TNF‐α, IL‐10) in the hippocampus of AD model rats to explore IPCO's potential mechanisms. One‐way ANOVA showed that the treatment factor had a highly significant main effect on SOD activity (*F* (4, 10) = 30.12, *p* < 0.0001, partial *η* ^2^ = 0.923), MDA level (*F* (4, 10) = 39.59, *p* < 0.0001, partial *η* ^2^ = 0.941), and GSH‐Px activity (*F* (4, 10) = 48.95, *p* < 0.0001, partial *η* ^2^ = 0.951). Specifically, the SOD activity in the model group (688.06 ± 12.37 U/g) was significantly lower than that in the control group (773.63 ± 8.14 U/g, *p* < 0.0001) and all IPCO intervention groups (LDG: 722.74 ± 7.52 U/g, *p* = 0.0116; MDG: 744.65 ± 13.72 U/g, *p* = 0.0003; HDG: 744.51 ± 6.13 U/g, *p* = 0.0003). In contrast, the MDA level and GSH‐Px activity in the model group (40.31 ± 2.23 nmol/g; 214.75 ± 8.05 nmol/min/g) were significantly higher than those in the control group (MDA: 28.64 ± 0.48 nmol/g, *p* < 0.0001; GSH‐Px: 160.53 ± 3.30 nmol/min/g, *p* < 0.0001) and IPCO‐treated groups (LDG: MDA 32.58 ± 1.10 nmol/g, *p* = 0.0001, GSH‐Px 195.90 ± 6.38 nmol/min/g, *p* = 0.0116; MDG: MDA 31.22 ± 0.54 nmol/g, *p* < 0.0001, GSH‐Px 182.88 ± 2.88 nmol/min/g, *p* = 0.0002; HDG: MDA 30.89 ± 0.94 nmol/g, *p* < 0.0001, GSH‐Px 166.75 ± 4.86 nmol/min/g, *p* < 0.0001) (Figure 5A–C). Regarding neuroinflammatory markers, the analysis detected significant overall effects of the treatment factor on IL‐10 (*F* (4, 10) = 410.11, *p* < 0.0001, partial *η* ^2^ = 0.994), IL‐6 (*F* (4, 10) = 292.63, *p* < 0.0001, partial *η* ^2^ = 0.992), IL‐1β (*F* (4, 10) = 128.35, *p* < 0.0001, partial *η* ^2^ = 0.981), and TNF‐α (*F* (4, 10) = 1752.92, *p* < 0.0001, partial *η* ^2^ = 0.999). The IL‐4 levels showed no significant difference between the model group (177.47 ± 1.32 pg/mL) and the low‐dose IPCO group (LDG: 171.13 ± 0.48 pg/mL, *p* > 0.05). The levels of IL‐6, IL‐1β, and TNF‐α in the model group were significantly higher than those in the medium‐ and high‐dose IPCO groups and the control group, while IL‐10 showed no significant difference between the model group (54.05 ± 0.80 pg/mL) and the low‐dose IPCO group (LDG: 56.18 ± 0.30 pg/mL, *p* > 0.05), but was significantly lower than in the medium‐dose IPCO group (MDG: 70.90 ± 0.72 pg/mL, *p* < 0.0001), high‐dose IPCO group (HDG: 74.74 ± 1.48 pg/mL, *p* < 0.0001), and control group (75.77 ± 0.72 pg/mL, *p* < 0.0001). Furthermore, the TNF‐α level was highest in the model group (379.07 ± 1.20 pg/mL) and lowest in the control group (293.52 ± 0.75 pg/mL), with a clear dose‐dependent decrease observed in the IPCO‐treated groups (LDG: 348.02 ± 1.64 pg/mL; MDG: 333.79 ± 1.33 pg/mL; HDG: 324.70 ± 1.40 pg/mL) (Figure 5D–H). All analyses were based on five experimental groups with a total sample size of *N* = 15.

Acetylcholinesterase (AChE), a key enzyme regulating acetylcholine hydrolysis, plays a dual role in AD pathology. It not only stably colocalizes with AD‐characteristic pathological markers, amyloid deposits, but also functions as a potent amyloidogenic promoter, exerting more significant pathological effects compared to other related proteins (Gajendra et al. 2024). To systematically evaluate AD progression across experimental groups, hippocampal AChE levels were quantitatively assessed by measuring hippocampal AChE concentrations. One‐way ANOVA showed that the treatment factor had a highly significant main effect on hippocampal AChE levels (*F*(4, 10) = 251.98, *p* < 0.0001, partial *η* ^2^ = 0.992). The results indicated that the AChE level was highest in the model group (0.0587 ± 0.0013 U/mgprot) and lowest in the control group (0.0340 ± 0.0008 U/mgprot), with a clear dose‐dependent reduction observed across IPCO treatment groups (LDG: 0.0570 ± 0.0014; MDG: 0.0499 ± 0.0012; HDG: 0.0447 ± 0.0003 U/mgprot) (Figure 5I,J). The analysis was based on five experimental groups with a total sample size of *N* = 15. In summary, chronic AlCl_3_ exposure successfully induced a series of phenotypes consistent with core AD features in Whistar rats, including increased AChE activity.

In summary, IPCO administration significantly ameliorated oxidative stress and neuroinflammation in AD model rats in a dose‐responsive manner, with higher doses producing more substantial improvements.

### 3.6 Determination of Intestinal Microbiota in Feces in Each Group

#### 3.6.1 Alpha and Beta Diversity and Differences in the Composition of Dominant Gut Microbiota

**FIGURE 6:**
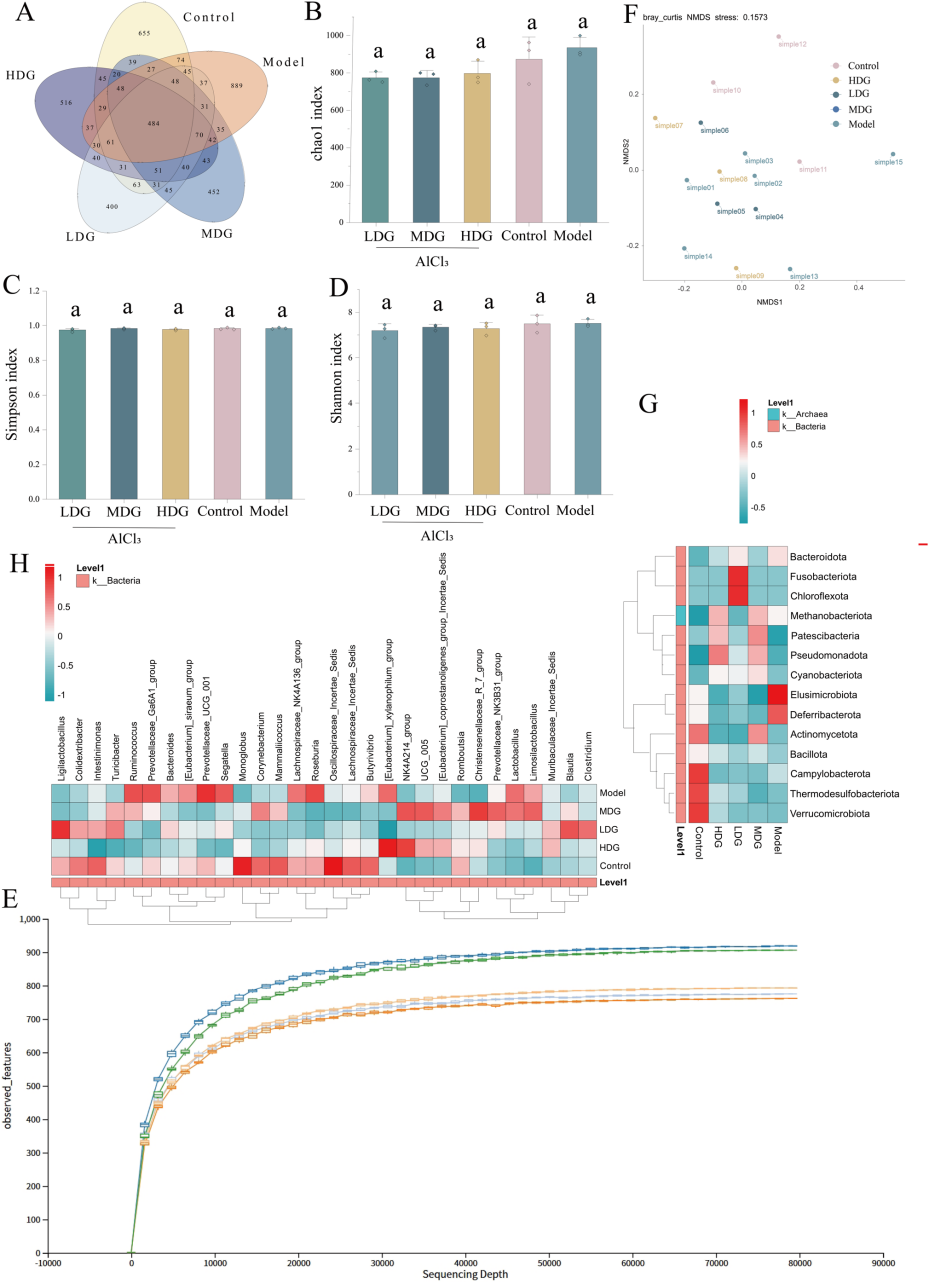
Effects of IPCO on intestinal flora diversity, specificity and generic level structural differences in Alzheimer's disease induced by aluminum chloride. Number of OTUs in different groups (A), Chao1 index (B), Simpson index (C), Shannon index (D), observed‐features sparse curve (E), NMD graph based on Bray‐Curtis heterogeneity (F), community composition of the gut microbiota at the phylum level (G), community composition of the gut microbiota at the genus level (H). Values are expressed as mean ± SD from independent replicates (*n* = 3). Statistical analysis was performed using one‐way ANOVA with Tukey's post hoc test. Values with different letters are significantly different (*p* < 0.05). The combination of letters (e.g., “ab”) indicates that the value is not significantly different from those marked with either “a” or “b”, with corresponding exact *p*‐val*ues detailed in the manuscrip*t. The Benjamini‐Hochberg method was used to correct the FDR, and a *p*‐value < 0.05 after FDR correction was used as the standard for statistical significance of the heatmap.

To assess gut microbiota composition and sequencing depth, we sequenced all samples with an average effective depth of 84,660 ± 6737 reads, exceeding typical thresholds for microbial diversity. Rarefaction curves confirmed that at 77,000 reads, further sequencing would yield negligible gains, supporting the depth for alpha‐ and beta‐diversity analyses (Figure 6E). Statistical analysis of microbial richness (Chao1 index) and diversity (Shannon and Simpson indices) showed no significant differences between groups (all *p* > 0.05), indicating stable diversity (Figure 6B–D). Venn analysis identified 484 core OTUs shared among all groups, with unique OTUs varying across groups (Control: 655, Model: 889, LDG: 400, MDG: 452, HDG: 516) (Figure 6A). The Model group had the highest total OTUs, while IPCO‐treated groups showed a dose‐dependent reduction, suggesting microbiota modulation to alleviate lipid metabolic dysfunction. OTU abundance was lowest in LDG (6787), higher in HDG (13221), and intermediate in Model (7452), Control (8780), and MDG (8103). HDG shared the highest proportion of OTUs with other groups, indicating that high‐dose IPCO may remodel microbial niches while maintaining overall diversity. β‐diversity analysis based on Bray‐Curtis distance showed clear separation between IPCO‐treated groups and the Model/Control groups (Figure 6F).

Analyses at the phylum‐ (14 taxa) and genus‐level (top 30 taxa), subjected to rigorous multiple comparison correction (pairwise comparisons with FDR adjustment following significant Kruskal‐Wallis tests, FDR < 0.05), revealed structural shifts in the gut microbiota (Figure 6G,H). At the genus level, the Model group was dominated by *[Eubacterium]‐siraeum‐group* (*Firmicutes*), *Ruminococcus* (*Firmicutes*), *Prevotellaceae‐Ga6A1‐group* (*Bacteroidetes*), *Segatella* (*Bacteroidetes*), *Prevotellaceae‐UCG‐001* (*Bacteroidetes*), *Roseburia* (*Firmicutes*), *Bacteroides* (*Bacteroidetes*), *Lachnospiraceae‐NK4A136‐group* (*Firmicutes*), while the Control group uniquely enriched *Colidextribacter* (*Firmicutes*), *Intestinimonas* (*Firmicutes*), *Monoglobus* (*Firmicutes*), *Corynebacterium* (*Actinomycetota*), *Mammaliicoccus* (*Firmicutes*), *Oscillospiraceae‐Incertae‐Sedis* (*Firmicutes*), *Lachnospiraceae‐Incertae‐Sedis* (*Firmicutes*), *Butyrivibrio* (*Firmicutes*). IPCO intervention induced dose‐specific effects: HDG promoted *[Eubacterium]‐xylanophilum‐group* (*Firmicutes*) and *NK4A214‐group* (*Firmicutes*); LDG increased *Ligilactobacillus* (*Firmicutes*)、*Blautia* (*Firmicutes*) and *Clostridium* (*Firmicutes*); MDG upregulated *Prevotellaceae‐NK3B31‐group* (*Bacteroidetes*), *Christensenellaceae‐R‐7‐group* (*Firmicutes*), *Limosilactobacillus* (*Firmicutes*), *[Eubacterium]‐coprostanoligenes‐group‐Incertae‐Sedis* (*Firmicutes*), *UCG‐005* (*Bacteroidetes*). At the phylum level, Model group exhibited elevated *Elusimicrobiota* And *Deferribacterota*, whereas Control group was enriched with *Actinomycetota*, *Campylobacterota*, *Thermodesulfobacteriota*, and *Verrucomicrobiota*. IPCO treatments differentially modulated phyla: HDG enhanced *Pseudomonadota*; LDG activated *Chloroflexota* and *Fusobacteriota*. These findings confirm that IPCO dose‐dependently reshapes gut microbiota in AD model rats.

#### 3.6.2 The Composition of Intestinal Microbiota Was Significantly Different Among the Groups

**FIGURE 7:**
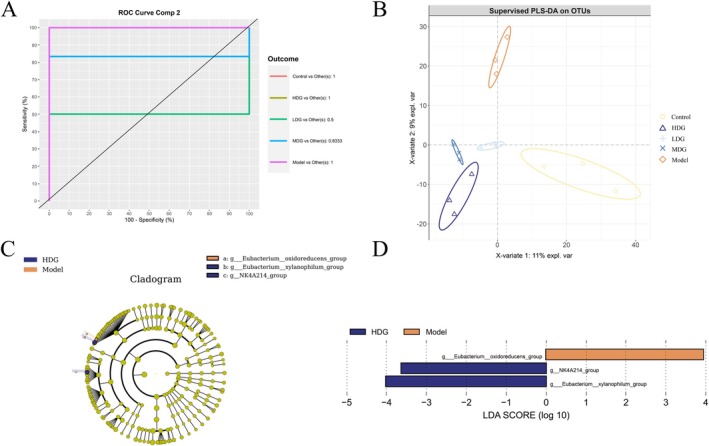
Phylogenetic distribution of functional biomarkers in each group. PLS‐DA ROC curves of OTU for each group (A), PLS‐DA coordinates of each OTU (B), LEfSe analyzes the cladogram graph (C), LEfSe analyzed the LDA histogram (D). Values are expressed as mean ± SD from independent replicates (*n* = 3). The LEfSe method uses the Benjamini‐Hochberg procedure for false discovery rate (FDR) correction, with the significance threshold set at *p* < 0.05 after FDR correction.

Multivariate analysis using Partial Least Squares Discriminant Analysis (PLS‐DA) applied to operational taxonomic unit (OTU) profiles demonstrated significant gut microbial community restructuring following IPCO administration, exhibiting dose‐responsive gradient separation among experimental cohorts. Notably, IPCO‐treated groups exhibited proximal spatial distances to the Control group in PLS‐DA plots, suggesting their potential to modulate microbial communities toward a healthier state (Figure 7A,B). To further investigate the regulatory mechanisms of AD and dose–response differences, we systematically compared the microbial compositions among the Control, Model, and dose‐treated groups using LEfSe (Linear Discriminant Analysis Effect Size) multilevel discriminant analysis. This analysis applied stringent criteria (LDA score > 2.0 and an FDR‐adjusted *p* < 0.05) to identify microbial genera/ASVs with significant differential abundance between groups. The analysis successfully identified characteristic biomarkers for the Model and HDG groups. Compared to other groups, the Model group showed significant enrichment of the *[Eubacterium]‐oxidoreducens‐group* (*Firmicutes*), while the HDG group was uniquely enriched for the *[Eubacterium]‐xylanophilum‐group* (*Firmicutes*) and the *NK4A214‐group* (*Firmicutes*) (Figure 7C,D). These biomarkers not only provide clues for understanding the association between IPCO's impact on specific bacterial genera and the alleviation of AD pathology but also clarify its dose‐effect relationships, offering critical theoretical support for precision nutrition strategies targeting the gut microbiota.

### 3.7 Determination of SCFAs in Feces

SCFAs can influence the pathological process of AD through various mechanisms, including epigenetic regulation, regulation of neuroinflammation, maintenance of blood–brain barrier homeostasis, promotion of brain metabolic balance, and inhibition of amyloid protein deposition. Therefore, intestinal SCFA levels have been proposed as a potential biomarker for AD progression (Chen, Meng, and Shen 2022). In this study, the concentrations of seven SCFAs (acetic acid, butyric acid, hexanoic acid, isobutyric acid, isovaleric acid, propionic acid, and valerate) in the feces of five groups of animals: model group, control group, and low‐, medium‐, and high‐dose IPCO intervention groups (LDG, MDG, HDG). One‐way ANOVA showed that the effects of treatment factors on different SCFAs varied. Specifically, the treatment factors had extremely significant main effects on the levels of acetic acid (*F* (4, 10) = 326.93, *p* < 0.0001, partial *η* ^2^ = 0.992), butyric acid (*F* (4, 10) = 278.20, *p* < 0.0001, partial *η* ^2^ = 0.991), and propionic acid (*F* (4, 10) = 153.99, *p* < 0.0001, partial *η* ^2^ = 0.984). However, the treatment factors did not show a significant main effect on the levels of hexanoic acid (*F* (4, 10) = 2.20, *p* = 0.1426, partial *η* ^2^ = 0.468), isobutyric acid (*F* (4, 10) = 0.066, *p* = 0.9908, partial *η* ^2^ = 0.026), isovaleric acid (*F* (4, 10) = 0.590, *p* = 0.6773, partial *η* ^2^ = 0.191), and valerate (*F* (4, 10) = 0.880, *p* = 0.5097, partial *η* ^2^ = 0.260) (all *p* > 0.05) (Table 4). This analysis was based on 5 experimental groups with a total sample size of *N* = 15. The results showed a clear dose–response relationship after IPCO intervention, with acetic acid, butyric acid, and propionic acid levels increasing in a dose‐dependent manner with increasing dose. Specifically, the high‐dose IPCO group (HDG: 3226.79 ± 72.48; 1365.41 ± 47.26; 813.26 ± 10.97 μg/g) had significantly higher levels of these three SCFAs than the other two IPCO intervention groups (LDG: 2369.31 ± 36.93; 699.50 ± 8.17; 543.01 ± 27.60 μg/g; *p* < 0.0001; MDG: 2515.95 ± 80.42, *p* < 0.0001; 987.90 ± 58.27, *p* < 0.0001; 665.94 ± 11.46 μg/g, *p* = 0.0002), and also significantly higher than the control group (2149.23 ± 16.25; 645.77 ± 3.95; 470.24 ± 35.03 μg/g; *p* < 0.0001) and the model group (1754.87 ± 12.27; 473.29 ± 29.84; 344.92 ± 30.29 μg/g; *p* < 0.0001) were dose‐dependently increased by IPCO in AD model animals, suggesting that it may exert a potential protective effect in AD by regulating gut microbiota‐derived metabolites.

**TABLE 4:** Concentration of SCFAs in feces of rats in each group.

Sample name	LDG	HDG	Control	Model	MDG	*F*‐value (df = 4, 10)	*p*	partial *η* ^2^
Acetic acid (μg/g)	2369.31 ± 36.93^c^	3226.79 ± 72.48^a^	2149.23 ± 16.25^d^	1754.87 ± 12.27^e^	2515.95 ± 80.42^b^	326.930	< 0.0001	0.992
Butyric acid (μg/g)	699.50 ± 8.17^c^	1365.41 ± 47.26^a^	645.77 ± 3.95^c^	473.29 ± 29.84^d^	987.90 ± 58.27^b^	278.200	< 0.0001	0.991
Caproic acid (μg/g)	26.46 ± 7.21^a^	39.39 ± 16.98^a^	46.13 ± 22.07^a^	16.39 ± 19.91^a^	15.95 ± 5.83^a^	153.99	< 0.0001	0.984
Isobutyric acid (μg/g)	37.78 ± 1.96^a^	37.37 ± 13.96^a^	38.85 ± 19.03^a^	34.66 ± 11.75^a^	34.99 ± 7.98^a^	2.200	0.1426	0.468
Isovaleric acid (μg/g)	21.39 ± 3.07^a^	21.61 ± 10.13^a^	30.02 ± 19.05^a^	22.87 ± 5.42^a^	17.84 ± 2.32^a^	0.066	0.9908	0.026
Propionic acid (μg/g)	543.01 ± 27.60^c^	813.26 ± 10.97^a^	470.24 ± 35.03^d^	344.92 ± 30.29^e^	665.94 ± 11.46^b^	0.590	0.6773	0.191
Valeric acid (μg/g)	55.00 ± 6.19^a^	74.94 ± 27.48^a^	64.04 ± 26.57^a^	49.59 ± 19.60^a^	48.9 ± 13.53^a^	0.880	0.5097	0.260

*Note:* Values with different superscript letters in the same row indicate significant differences (*p* < 0.05) among treatments. The combination of letters (e.g., “ab”) denotes no significant difference from groups marked with either “a” or “b”. The results are the means ± SD (*n* = 3). *F*‐value (df = 4, 10) denotes the *F*‐statistic from one‐way ANOVA, with the numbers in parentheses representing the between‐groups and within‐groups degrees of freedom, respectively; *p* indicates the significance level; partial *η* ^2^ is the effect size, representing the proportion of variance explained by the treatment factor.

### 3.8 Correlation Analysis Between Gut Microbiota, SCFAs and Environmental Factors

**FIGURE 8:**
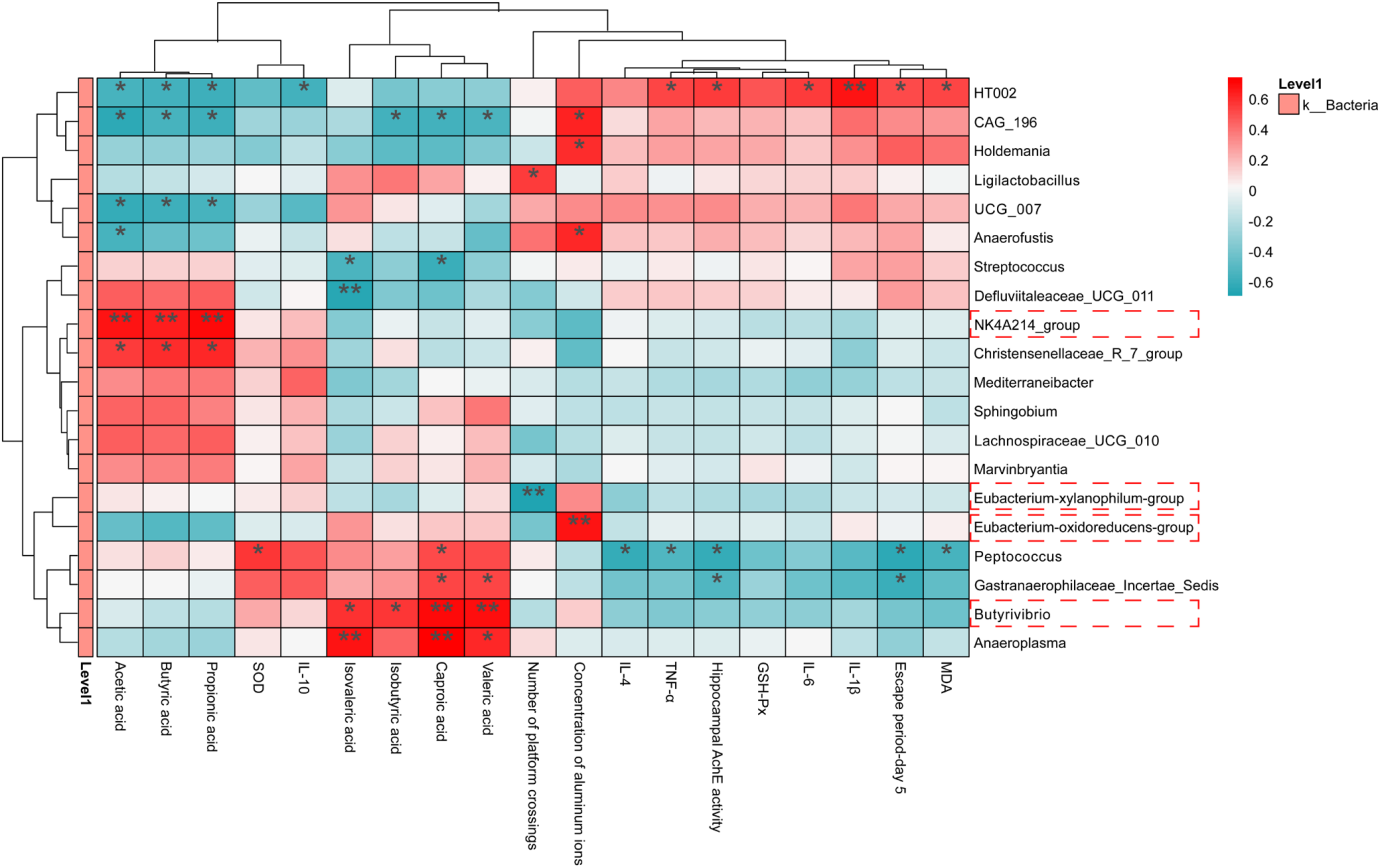
Heatmap showing Spearman correlation coefficients between gut microbiota and environmental factors. The asterisks (*, **, ***) represent the unadjusted nominal *p*‐value levels (< 0.05, < 0.01, < 0.001, respectively). For formal hypothesis testing, *p*‐values were adjusted for multiple comparisons using the Benjamini–Hochberg FDR procedure, and an FDR‐adjusted *p*‐value < 0.05 was set as the threshold for statistical significance.

To elucidate the potential mechanism by which IPCO modulates the gut microbiota to ameliorate AD progression, this study conducted a multidimensional correlation analysis of 18 key microbial genera significantly associated with environmental factors, along with HDG‐enriched groups. To investigate the associations between SCFA concentrations and behavioral scores, Spearman correlation analysis was performed, with all *p*‐values adjusted for multiple comparisons using the Benjamini‐Hochberg (FDR) correction (significance threshold: FDR < 0.05). This correction strategy was consistently applied in subsequent analyses integrating microbial abundance, SCFAs, and behavioral indices into correlation networks. The results showed that the core genus *[Eubacterium]‐oxidoreducens‐group* (*Firmicutes*) in the model group was significantly positively correlated with hippocampal aluminum ion concentration (FDR < 0.01). The HDG‐specific *[Eubacterium]‐xylanophilum‐group* (*Firmicutes*) showed a significant negative correlation with spatial memory (number of platform crossings, FDR < 0.01). The dominant genus *NK4A214‐group* (*Firmicutes*) was significantly positively correlated with acetic acid, butyric acid, and propionic acid (all FDR < 0.01). Further analysis revealed that Butyricicoccus was significantly correlated with hexanoic acid, isobutyric acid, valeric acid, and isovaleric acid (FDR < 0.05 or < 0.01), but not with butyric acid (FDR > 0.05) (Figure 8). It is important to emphasize that these findings represent statistical associations, and causal relationships remain to be confirmed through experiments such as fecal microbiota transplantation or SCFA supplementation. In summary, IPCO may be associated with the amelioration of AD‐related cognitive impairment through the modulation of specific gut microbiota and their SCFA metabolic pathways.

## 4 Discussion

### 4.1 A Proposed Mechanism: Potential Synergistic Modulation of AD Pathology via *PPARG* , *PTGS2* , and *ERBB2* by Candidate IPCO Components

Recent studies have highlighted IPCO's antioxidant properties, linked to unsaturated fatty acids and lipid‐soluble compounds (Zhang et al. 2023; Guo et al. 2023). Our GC–MS analysis confirmed these constituents and identified Beta‐amyrin. Based on this, we hypothesized IPCO's potential activity against AD pathology. To test this, we used network pharmacology and molecular docking, identifying linoleic acid, 2,4‐di‐tert‐butylphenol, and Beta‐amyrin as active components. Potential therapeutic targets included *PTGS2*, *PPARG*, *ESR1*, *AKT1*, *GSK3B*, *HIF1A*, *ERBB2*, and *ESR2*.

GO and KEGG enrichment analyses suggested IPCO's involvement in AD‐related pathways, such as calcium signaling, neuroactive ligand‐receptor interaction, and PPAR signaling (Chami 2021; Azargoonjahromi et al. 2024; Wójtowicz et al. 2020; Chen et al. 2024). Molecular docking indicated potential binding of these compounds to *PPARG*, *PTGS2*, and *ERBB2*. Linoleic acid and 2,4‐di‐tert‐butylphenol might interact with *PTGS2*, while Beta‐amyrin could bind *ERBB2*.

These findings suggest a model where IPCO components may influence AD through receptor modulation, signal transduction, and regulation of lipid homeostasis, neuroinflammation, and synaptic plasticity. However, experimental validation is required to confirm target engagement and pathway modulation.

### 4.2 High‐Dose IPCO Improves AD Better: Supported by Multi‐Index Data

This study aimed to comprehensively evaluate the effects of IPCO on an AlCl_3_‐induced AD rat model through multiple experimental approaches, including behavioral assessment, biochemical analysis, and elemental determination. Spatial learning and memory were evaluated using the escape latency on the final day of training and the number of platform crossings during the probe trial in the MWM (Puzzo et al. 2014). The results showed no significant differences among IPCO intervention groups in these behavioral indices, and no clear dose–effect relationship was observed. This lack of a linear behavioral response does not necessarily indicate insufficient efficacy of IPCO, but may instead reflect the complexity of neural repair processes, in which molecular and biochemical alterations may precede functional neural network stabilization and cognitive recovery (Murphy and Corbett 2009; Turrigiano 2008).

To exclude the potential confounding influence of dietary aluminum exposure, the aluminum content in the standard diet was quantified using ICP‐OES microwave digestion (Karasakal 2021), yielding a value of 75.72 ± 7.56 mg/kg. Based on previous estimates, the corresponding aluminum intake was approximately 1.64 mg/kg body weight (Martinez et al. 2017). Previous studies (Walton 2009) have shown that aluminum intake at comparable levels can elevate serum aluminum concentrations and promote hippocampal aluminum accumulation. In the present study, although the control group exhibited a relatively high baseline aluminum level in the hippocampus, it remained significantly lower than that observed in the AlCl_3_‐induced model group. Importantly, IPCO treatment resulted in a dose‐dependent reduction in hippocampal aluminum accumulation, with medium‐ and high‐dose groups restoring aluminum levels close to those of the control group. Had IPCO been ineffective, aluminum levels in treated groups would be expected to resemble those of the model group. These findings suggest that IPCO may contribute to mitigating aluminum overload induced by experimental exposure, although further confirmation is required.

Consistent with the aluminum burden findings, biochemical analyses revealed that IPCO treatment alleviated AD‐related deterioration, as reflected by improvements in oxidative stress markers (MDA, SOD, and GSH‐Px) and modulation of inflammatory cytokines (IL‐1β, IL‐6, TNF‐α, IL‐4, and IL‐10). These effects showed a potential dose‐dependent trend, with the HDG exhibiting the most pronounced biochemical improvements. In addition, AChE activity correlated with oxidative stress and inflammatory parameters, further supporting a neuroprotective role of IPCO, particularly at higher doses. Given the critical role of cortical acetylcholine in regulating cognitive stability and signal‐to‐noise ratio, such modulation may have important implications for network‐level function (Sarter and Bruno 1997).

Although biochemical markers showed significant improvement, the recovery of cognitive function did not follow a linear dose‐dependent pattern. Notably, the HDG exhibited a fluctuating recovery trajectory in the MWM test, showing no significant improvement compared to the model group on days 2–4, followed by a marked recovery on day 5. Comparable results across all groups in swimming speed, total distance, and visible platform tests indicated that this phenomenon was not caused by visual or motor deficits (Table S4). Furthermore, all groups demonstrated a stable increasing trend in body weight, with no significant difference between the HDG and the control group, ruling out non‐specific neurotoxicity of IPCO as the cause of the delayed behavioral response (Table S5). This dissociation between biochemical recovery and behavioral performance suggests that high‐intensity intervention may temporarily disrupt neural network dynamics. Neural function highly depends on the balance between excitation and inhibition (E/I balance); although benefits at the molecular level are evident, excessive excitatory drive—particularly through glutamatergic or cholinergic systems—has been proven to destabilize cortical circuits and impair information processing capacity, even when underlying molecular improvements are present (Yizhar et al. 2011; Anticevic et al. 2012). It is particularly important to emphasize that one of the core objectives of this study was to establish an aluminum exposure model relevant to the pathogenesis of AD, rather than a broad neurotoxicity model. The dose (100 mg/kg/day) and duration (48 days) we employed have clear precedent in AD research and are widely used to simulate a chronic aluminum load sufficient to promote AD‐like pathology but insufficient to cause acute organ failure. The validity of this model, referencing prior studies, is strongly supported by the induced, highly specific spectrum of AD‐like phenotypes: selective cognitive domain impairment, Aβ metabolic imbalance, and Tau protein pathology. Additionally, the finding that all treatment groups showed a stable increasing trend in body weight throughout the experimental period, with no significant difference between the high‐dose IPCO group and the control group, further excludes the possibility of non‐specific systemic or neurotoxicity caused by IPCO or the aluminum exposure protocol itself (Weng et al. 2020). This strengthens the conclusion that the observed behavioral and pathological changes are specific effects stemming from AD‐related mechanisms.

In addition to circuit‐level instability, fluctuating behavioral outcomes may also reflect dynamic shifts in behavioral control strategies. Cognitive task execution relies on the coordinated engagement of multiple memory systems, including the prefrontal cortex, hippocampus, and striatum. Under conditions of strong neuromodulatory influence or neural stress, behavioral control may shift from flexible, prefrontal cortex–dependent strategies toward more habitual, striatum‐mediated strategies, resulting in transient variability in task performance (Arnsten 2009). Competition between hippocampal‐ and striatum‐based memory systems during periods of heightened arousal has been well documented and provides a plausible explanation for the non‐linear behavioral recovery observed in the high‐dose IPCO group (Packard and Goodman 2012).

From a systems‐level perspective, these behavioral fluctuations may represent an adaptive phase of neural reorganization rather than a failure of treatment. Homeostatic plasticity mechanisms, such as synaptic scaling, are activated when neural activity is strongly perturbed, enabling gradual recalibration of network excitability and restoration of functional stability (Turrigiano 2008). This adaptive process aligns with the concept of hormesis, whereby intense but controlled biological stress may induce short‐term functional instability while ultimately promoting resilience and long‐term recovery (Calabrese and Mattson 2017). Accordingly, the delayed behavioral improvement observed in the high‐dose group may reflect a “rebuild after breakdown” process, in which transient imbalance precedes more stable cognitive restoration.

In conclusion, IPCO appears to exert multidimensional protective effects in the AlCl_3_‐induced AD rat model by reducing hippocampal aluminum accumulation, alleviating oxidative stress, suppressing neuroinflammation, and modulating AChE activity in a dose‐dependent manner. The absence of a linear behavioral dose–response relationship and the fluctuating recovery observed at high doses are more likely to reflect adaptive neural dynamics during the repair process rather than diminished therapeutic efficacy. These findings suggest that the optimal efficacy of IPCO may depend on precise control of dosage, treatment duration, and the timing of behavioral training, underscoring the importance of considering neural network stability and adaptive plasticity when evaluating cognitive outcomes.

In addition, by exploring the gut‐brain axis regulatory mechanisms, this study comprehensively investigated the potential pathways through which IPCO intervenes in AD. LEfSe analysis demonstrated significant enrichment of the barrier‐disrupting bacterial genus *[Eubacterium]‐oxidoreducens‐group* (*Firmicutes*) in the Model group, which has been shown to elevate lipopolysaccharide (LPS) levels, damage the intestinal epithelial barrier and blood–brain barrier, and exacerbate neuroinflammation (Zhang et al. 2020; Banks et al. 2015). Notably, the HDG exhibited specific enrichment of two neuroprotective SCFAs‐producing bacterial taxa—*[Eubacterium]‐xylanophilum‐group* (*Firmicutes*) and *NK4A214‐group* (*Firmicutes*)—whose metabolites, SCFAs, have been reported to regulate neuroinflammation and ameliorate AD pathology in previous studies (Zhuge et al. 2021; Shen et al. 2022). Environmental factor correlation analysis further revealed that the abundance of *[Eubacterium]‐oxidoreducens‐group* (*Firmicutes*) was significantly positively correlated with hippocampal aluminum accumulation, suggesting its potential role in metal toxicity‐mediated neural injury. In contrast, the abundance of *[Eubacterium]‐xylanophilum‐group* (*Firmicutes*) showed a significant negative correlation with the number of platform crossings in the Morris water maze, implying its influence on cognitive function via spatial memory‐related pathways. Furthermore, the abundance of the *NK4A214‐group* (*Firmicutes*) was significantly positively correlated with the intestinal SCFAs acetate, butyrate, and propionic acid, suggesting a key role in maintaining inflammatory homeostasis. Targeted SCFA metabolomics analysis showed that although there was no statistical difference in total SCFA levels among the groups, the combined regulatory effect of six SCFAs (acetate, propionate, butyrate, isobutyrate, valerate, and hexanoate) (except isovalerate) was significantly superior to that of the model group. These results suggest that high‐dose IPCO may improve AD pathology through a dual regulatory mechanism: inhibiting the proliferation of pro‐inflammatory *[Eubacterium]‐oxidoreducens‐group* (*Firmicutes*) while enriching the functional bacteria that produce SCFAs, exerting neuroprotective effects through metabolic network regulation.

### 4.3 The Neural Bifacial Nature of Linoleic Acid: From Metabolic Controversy to Decryption of Conformation‐Specific Anti‐AD Mechanisms

The current academic debate regarding the regulatory effects of dietary LA on AD pathology remains unresolved. Traditional perspectives posit that LA participates in pro‐inflammatory responses via the arachidonic acid (AA) metabolic axis (Blake et al. 2025), suggesting that excessive intake may exacerbate neuroinflammatory processes. However, recent population studies have demonstrated no significant correlation between LA intake and tissue AA accumulation levels in Western dietary contexts (Rett and Whelan 2011), indicating that the pro‐inflammatory effects of LA metabolites are subject to complex dose‐ and environment‐dependent modulation. Notably, LA isomers exhibit markedly distinct biological effects: for instance, *cis*‐9, *trans*‐11‐conjugated linoleic acid (CLA) exerts neuroprotective properties by reducing Aβ deposition and suppressing microglial proliferation (Fujita et al. 2021). This highlights the critical dependence of LA's biological impact on its spatial configuration and metabolic microenvironment. The present study identified (Z,Z)‐9,12‐octadecadienoic acid as the predominant LA form in IPCO, a configuration previously shown to mitigate Aβ25‐35‐induced hippocampal neuronal damage in mice (Zeng et al. 2023), thereby mechanistically corroborating the anti‐AD effects observed in this research. Collectively, these findings underscore that structural specificity analysis of LA and dynamic monitoring of its metabolic networks represent pivotal approaches for elucidating its dual neuroprotective/neurotoxic roles. In summary, these findings highlight that structural specificity analysis of LA and dynamic monitoring of its metabolic network are crucial for elucidating its dual neuroprotective/neurotoxic effects. Specifically, HDG demonstrated the most beneficial effect in improving AD, though future studies are needed to identify the critical turning point at which it exerts these positive effects.

### 4.4 Limitations and Future Prospects

While this study provides multi‐faceted evidence for IPCO's anti‐AD potential and proposes a preliminary synergistic mechanism model, the following limitations must be acknowledged. These limitations also delineate clear directions for future research.

First, at the level of mechanistic validation, the direct “compound‐target” interaction chain remains incompletely verified. Computationally predicted interactions between core constituents (e.g., linoleic acid, beta‐amyrin) and key targets (e.g., *PTGS2*, *PPARG*) require experimental confirmation through techniques such as enzyme activity assays, reporter gene analysis, and surface plasmon resonance. The causal relationship linking specific constituents, target modulation, and in vivo effects has not been established. Moreover, findings regarding the gut microbiota–SCFAs axis are descriptive and relational; observed associations between specific microbiota, SCFA levels, and behavioral phenotypes do not imply causality and may be influenced by unmeasured confounders. Future interventional studies—such as fecal microbiota transplantation, supplemental SCFA experiments, or longitudinal cohort studies—are needed to validate potential causal links.

Second, there are technical constraints in the experimental design and exploration of pathological mechanisms. The relatively small sample size (*n* = 8) combined with model heterogeneity may reduce statistical power. Key AD biomarkers (e.g., CSF Aβ42/p‐tau, plasma NfL) were not assessed, and oxidative stress measures were incomplete. Furthermore, serum aluminum levels were not measured, preventing exploration of IPCO's potential effect on aluminum load, a significant environmental risk factor. The exclusive use of male animals also limits the generalizability of the findings across sexes. Furthermore, for the measurement of aluminum content, oxidative stress markers, inflammatory cytokines, and acetylcholinesterase activity in the hippocampus, the sample size was further reduced to *n* = 3, which may affect the statistical robustness of the results. Although we sought to mitigate these limitations through rigorous experimental design and data analysis, the reduced sample size may still fail to fully capture broader variability within the experimental group. Moreover, although all animals were of the same strain, age, and sex and were housed under strictly controlled specific pathogen‐free (SPF) conditions, the selection of only three animals may not fully represent the characteristics of the entire experimental group. Therefore, future studies should consider increasing the sample size and refining the experimental design to enhance the generalizability and reliability of the findings.

Third, pharmacological and safety assessments are insufficient. The lack of absolute quantification of specific IPCO constituents and pharmacokinetic data precludes precise analysis of dose–response relationships for individual compounds. Safety evaluation is particularly incomplete, relying only on indirect measures such as body weight and behavior without direct evidence from serum biochemistry (e.g., liver/kidney function markers) or histopathology. This constrains definitive conclusions on IPCO's potential organ toxicity, especially at high doses associated with the “biphasic effect.”

To establish causal mechanisms and promote clinical translation, future research should focus on three key aspects: First, computational predictions should be experimentally validated through methods such as enzyme activity assays, reporter gene analysis, and Western blotting, while compound‐target binding interactions should be directly measured using techniques like surface plasmon resonance. Second, pharmacokinetic‐pharmacodynamic (PK‐PD) studies should be conducted to assess blood–brain barrier penetration and target tissue distribution of active ingredients. These studies must systematically incorporate comprehensive AD biomarkers, oxidative stress indicators, and key exposure parameters such as serum aluminum levels to enhance the reliability and clinical relevance of the findings. Third, expanding sample sizes, implementing multi‐center validation, and including female animal models will significantly improve the generalizability and statistical power of the results. Finally, it must be emphasized that systematic preclinical toxicity assessment is as critical as confirming core drug efficacy and represents an indispensable step in advancing translational research.

## 5 Conclusion

This study suggests that the core candidate constituents of IPCO— (Z,Z)‐9,12‐octadecadienoic acid, 2,4‐di‐tert‐butylphenol, and β‐amyrin—may influence AD pathological progression through potential multi‐target synergy. Based on computational modeling, molecular docking analyses predict that these constituents could interact with potential targets such as *PTGS2*, *PPARG*, and *ERBB2*, possibly modulating neuroinflammation, lipid metabolism, and calcium signaling pathways. In vivo results indicate an association between IPCO intervention and improvements in cognitive function (shortened escape latency in the Morris water maze), reduced hippocampal aluminum burden, and upregulation of the anti‐inflammatory cytokine IL‐10. At the gut microbiome level, microbiota analysis of the IPCO‐treated groups showed an increased relative abundance of putative SCFA‐producing genera (e.g., *[Eubacterium]‐xylanophilum‐group* and *NK4A214‐group*), correlating with a rising trend in the concentrations of butyrate and other SCFAs. It is particularly noteworthy that, while IPCO exhibited potential anti‐AD effects across multiple dimensions with the high‐dose group showing the most pronounced improvements in several metrics, this “optimal efficacy” is derived from a composite assessment of indicators. The study observed complex, dose‐dependent variations in some cognitive measures, underscoring the imperative for future pharmacokinetic studies to precisely define its therapeutic window and to elucidate the mechanisms underlying its potential bidirectional regulatory effects. This research provides preliminary multi‐omics evidence and a theoretical hypothesis for expanding the functional applications of IPCO, the clinical translation potential of which awaits further in‐depth mechanistic validation and safety assessment.

## Author Contributions

**Weijie Chang:** conceptualization, data curation, investigation, methodology, resources, visualization, writing – original draft. **Xiufang Huang:** writing – review and editing, formal analysis, software. **Yaobing Chen:** data curation. **Kai Luo:** funding acquisition, project administration, writing – review and editing. **Jianquan Kan:** writing – review and editing, funding acquisition.

## Funding

This work received funding from the National Natural Science Foundation of China (32472256), the Natural Science Foundation of Hubei Province (2023AFD080), The Open Fund of Hubei Engineering Research Centre of selenium food nutrition and health intelligent technology (Hubei Minzu University) (PT082401), The Open Fund of Hubei Provincial Key Laboratory of Occurrence and Intervention of Rheumatic Diseases (Hubei Minzu University) (PT022208), Hubei Key Laboratory of Biologic Resources Protection and Utilization (Hubei Minzu University) (KYPT012402).

## Ethics Statement

All animal studies were performed in accordance with the guidelines of the Animal Care and Use Committee (IACUC) of the Animal Experiment Center of Enshi Prefecture Central Hospital (approval number: 202408003).

## Conflicts of Interest

The authors declare no conflicts of interest.

## Supporting information


**Figure S1:** Total ion map of *Idesia polycarpa* crude oil.
**Table S1:** Power analysis.
**Table S2:** Rats maintain feed composition.
**Table S3:** Determination of aluminum ion concentration in feed.
**Table S4:** Analysis of swimming speed, total distance, and visible platform performance in the MWM experiment.
**Table S5:** Changes in body weight during the experiment.

## Data Availability

All data associated with this study are available from the corresponding author upon reasonable request.
