# Supplementary material for: Integrated Network Pharmacology, Molecular Docking, and Experimental Validation Elucidating the Therapeutic Mechanism of Idesia polycarpa Crude Oil in Aluminum Chloride‐Induced Alzheimer's Rat Models
Source: Food Sci Nutr. 2026 Apr 20;14(4):e71702. doi: 10.1002/fsn3.71702 (PMC13093899; doi:10.1002/fsn3.71702)
Supplement: Supplementary file 1 — Figure S1: Total ion map of Idesia polycarpa crude oil. Table S1: Power analysis. Table S2: Rats maintain feed composition. Table S3: Determination of aluminum ion concentration in feed. Table S4: Analysis of swimming speed, total distance, and visible platform performance in the MWM experiment. Table S5: Changes in body weight during the experiment. [file FSN3-14-e71702-s001.docx]

**Integrated network pharmacology, molecular docking, and experimental validation elucidating the therapeutic mechanism of *Idesia polycarpa* crude oil in Aluminum Chloride-Induced Alzheimer's rat models**





Figure S1: Total ion map of *Idesia polycarpa* crude oil.

Table S1. Power Analysis

| Index | Alpha value (type I error) | Beta value (type II error) | Power value (1-Beta value) | Group 1 sample size (n1) | Group 2 sample size (n2) | Sample size ratio (n1/n2) |
| --- | --- | --- | --- | --- | --- | --- |
| Morris water maze | 0.05 | 0.200 | 0.800 | 2.000 | 2.000 | 1 |
| Aluminum content | 0.05 | 0.200 | 0.800 | 3.000 | 3.000 | 1 |
| AChE activity | 0.05 | 0.200 | 0.800 | 3.000 | 3.000 | 1 |
| TNF-α | 0.05 | 0.200 | 0.800 | 2.000 | 2.000 | 1 |
| IL-6 | 0.05 | 0.200 | 0.800 | 2.000 | 2.000 | 1 |

Note: This table is derived from the study data of Liu et al., and is derived by SPSSAU.

Table S2. Rats maintain feed composition

| Index | Numeric value |
| --- | --- |
| Energy, kJ/100 g | 340 |
| Moisture, g/kg | ≤100 |
| Crude protein, g/kg | ≥180 |
| Crude fat, g/kg | ≥40 |
| Fiber, g/kg | ≤50 |
| Crude ash, g/kg | ≤80 |
| Calcium, g/kg | 10 -18 |
| Total phosphorus, g/kg | 6 -12 |
| Calcium: phosphorus, g/kg | 1.2:1-1.7:1 |

Note: This table is provided by Liaoning Changsheng bioengineering Co., Ltd

Table S3. Determination of aluminum ion concentration in feed

| Sample number | Sample mass m_0_ (g) | Constant volume V_0_ (mL) | Test elements | Test solution elemental concentration C_o_ (mg/L) | Dilution multiple (f) | Digestion solution element concentration C_1_ (mg/L) | Sample elemental content C_x_ (mg/kg) | Sample elemental content (%) |
| --- | --- | --- | --- | --- | --- | --- | --- | --- |
| 1 | 0.5085 | 25 | Al | 1.3123 | 1 | 1.3123 | 64.5164 | 0.0065% |
| 1 | 0.5085 | 25 | Al | 1.3565 | 1 | 1.3565 | 66.6900 | 0.0067% |
| 1 | 0.5085 | 25 | Al | 1.3672 | 1 | 1.3672 | 67.2189 | 0.0067% |
| 2 | 0.5107 | 25 | Al | 1.5875 | 1 | 1.5875 | 77.7130 | 0.0078% |
| 2 | 0.5107 | 25 | Al | 1.5801 | 1 | 1.5801 | 77.3473 | 0.0077% |
| 2 | 0.5107 | 25 | Al | 1.6128 | 1 | 1.6128 | 78.9501 | 0.0079% |
| 3 | 0.5041 | 25 | Al | 1.6580 | 1 | 1.6580 | 82.2252 | 0.0082% |
| 3 | 0.5041 | 25 | Al | 1.6884 | 1 | 1.6884 | 83.7349 | 0.0084% |
| 3 | 0.5041 | 25 | Al | 1.6760 | 1 | 1.6760 | 83.1203 | 0.0083% |
| Average value | 75.7240 ± 7.5639 | | | | | | | |

Note: Test conditions: pump rate: 60 r/min; plasma gas: 12.0 L/min; nebulizer flow: 0.70 L/min; stable time: 20 s; auxiliary gas: 1.0 L/min; reading access time: 5 s; sample flush Time: 20 s; RF power: 1250 w. The results are the means ± SD (n = 3).

Table S4. Analysis of swimming speed, total distance, and visible platform performance in the MWM experiment

| Training days | | Test Project | Control | Model | LDG | MDG | HDG | F-value (df=4,35) | *p*-value | partial η² |
| --- | --- | --- | --- | --- | --- | --- | --- | --- | --- | --- |
| Day 1 | | Swimming speed | 194.4 ± 3.4^a^ | 194.1 ± 3.1^a^ | 194.4 ± 3.1^a^ | 194.1 ± 3.2^a^ | 194.8 ± 2.8^a^ | 0.089 | 0.985 | 0.01 |
|  |  | Total distance | 9593.8 ± 350.4^c^ | 18057.5 ± 320.6^a^ | 14516.3 ± 2500.2^b^ | 11112.5 ± 280.4^c^ | 16367.5 ± 315.8^b^ | 90.460 | < 0.0001 | 0.912 |
| Day 2 | | Swimming speed | 194.7 ± 2.6^a^ | 194.7 ± 2.7^a^ | 194.2 ± 2.7^a^ | 194.7 ± 2.7^a^ | 195.3 ± 2.1^a^ | 0.077 | 0.989 | 0.009 |
|  |  | Total distance | 6153.8 ± 450.2^e^ | 16822.5 ± 310.4^a^ | 10762.5 ± 350.6^c^ | 9131.3 ± 220.8^d^ | 15201.3 ± 450.5^b^ | 1272.46 | < 0.0001 | 0.993 |
| Day 3 | | Swimming speed | 195.8 ± 2.6^a^ | 195.3 ± 2.5^a^ | 195.2 ± 2.6^a^ | 195.4 ± 2.6^a^ | 196.0 ± 2.2^a^ | 0.077 | 0.989 | 0.009 |
|  |  | Total distance | 4501.3 ± 480.3^e^ | 16032.5 ± 320.7^a^ | 9615.0 ± 240.4^c^ | 7411.3 ± 210.5^d^ | 14221.3 ± 220.6^b^ | 2014.15 | < 0.0001 | 0.996 |
| Day 4 | | Swimming speed | 196.2 ± 2.6^a^ | 195.4 ± 2.5^a^ | 195.4 ± 2.6^a^ | 195.4 ± 2.6^a^ | 195.4 ± 2.2^a^ | 0.077 | 0.989 | 0.009 |
|  |  | Total distance | 3011.3 ± 310.4^e^ | 15093.8 ± 305.2^a^ | 8431.3 ± 310.5^c^ | 6433.8 ± 310.8^d^ | 13233.8 ± 210.7^b^ | 2681.45 | < 0.0001 | 0.997 |
| Day 5 | | Swimming speed | 195.4 ± 2.6^a^ | 194.6 ± 2.6^a^ | 194.6 ± 2.9^a^ | 194.6 ± 3.1^a^ | 195.5 ± 2.6^a^ | 0.082 | 0.987 | 0.009 |
|  |  | Total distance | 2000.0 ± 180.2^c^ | 14457.5 ± 350.4^a^ | 6345.0 ± 1950.5^b^ | 5116.3 ± 1250.4^b^ | 4608.8 ± 250.6^b^ | 164.860 | < 0.0001 | 0.950 |
| Visible platform experiment | Latency | | 8.2 ± 1.1^a^ | 9.8 ± 1.5^a^ | 9.1 ± 1.2^a^ | 8.7 ± 1.4^a^ | 8.5 ± 1.3^a^ | 1.470 | > 0.05 | 0.144 |
|  | swimming speed | | 201.5 ± 12.4^a^ | 194.8 ± 15.2^a^ | 198.2 ± 10.8^a^ | 202.1 ± 13.5^a^ | 199.6 ± 14.2^a^ | 0.043 | > 0.05 | 0.005 |

Note: Values with different superscript letters in the same row indicate significant differences (*p* < 0.05) among treatments. The combination of letters (e.g., "ab") denotes no significant difference from groups marked with either "a" or "b". The results are the means ± SD (n = 8). F-value (df=4,35) denotes the F-statistic from one-way ANOVA, with the numbers in parentheses representing the between-groups and within-groups degrees of freedom, respectively; *p* indicates the significance level; partial η² is the effect size, representing the proportion of variance explained by the treatment factor.

Table S5. Changes in body weight during the experiment

| Time (Week) | Control | Model | LDG | MDG | HDG | F-value (df=4,35) | *p*-value | partial η² |
| --- | --- | --- | --- | --- | --- | --- | --- | --- |
| Week 0 | 230.5 ± 6.3^a^ | 230.7 ± 5.9^a^ | 230.7 ± 6.2^a^ | 230.6 ± 6.5^a^ | 231.0 ± 5.8^a^ | 0.007 | 0.9999 | 0.001 |
| Week 1 | 236.4 ± 6.1^a^ | 231.1 ± 5.8b | 232.1 ± 6.3^ab^ | 233.5 ± 6.5^ab^ | 235.2 ± 6.2^a^ | 6.700 | 0.0004 | 0.434 |
| Week 2 | 243.2 ± 6.5^a^ | 233.4 ± 5.5^c^ | 235.4 ± 5.9^b^ | 237.2 ± 6.2^ab^ | 240.5 ± 6.4^a^ | 29.774 | < 0.0001 | 0.773 |
| Week 3 | 249.8 ± 6.8^a^ | 236.2 ± 5.2^c^ | 238.6 ± 6.1^b^ | 241.1 ± 6.0^b^ | 246.8 ± 6.7^a^ | 72.995 | < 0.0001 | 0.893 |
| Week 4 | 256.5 ± 7.1^a^ | 238.5 ± 4.9^c^ | 241.2 ± 5.8^b^ | 244.5 ± 6.3^b^ | 253.1 ± 6.5^a^ | 143.151 | < 0.0001 | 0.942 |
| Week 5 | 263.1 ± 6.9^a^ | 239.6 ± 4.5^c^ | 243.8 ± 6.2^b^ | 247.2 ± 6.4^b^ | 260.4 ± 6.8^a^ | 245.375 | < 0.0001 | 0.966 |
| Week 6 | 270.4 ± 7.2^a^ | 243.8 ± 4.3^d^ | 246.1 ± 6.5^c^ | 250.4 ± 6.7^b^ | 268.2 ± 6.9^a^ | 448.707 | < 0.0001 | 0.981 |
| Week 7 | 275.2 ± 7.4^a^ | 244.2 ± 4.1^d^ | 248.5 ± 6.3^c^ | 252.6 ± 6.5^b^ | 274.5 ± 7.2^a^ | 834.306 | < 0.0001 | 0.990 |
| Day 60 | 279.1 ± 4.2^a^ | 246.5 ± 2.8^c^ | 250.4 ± 5.1^b^ | 254.8 ± 5.5^b^ | 278.4 ± 3.9^a^ | 875.373 | < 0.0001 | 0.990 |

Note: Values with different superscript letters in the same row indicate significant differences (*p* < 0.05) among treatments. The combination of letters (e.g., "ab") denotes no significant difference from groups marked with either "a" or "b". The results are the means ± SD (n = 8). F-value (df=4,35) denotes the F-statistic from one-way ANOVA, with the numbers in parentheses representing the between-groups and within-groups degrees of freedom, respectively; *p* indicates the significance level; partial η² is the effect size, representing the proportion of variance explained by the treatment factor.
